# A biology-based quality-diversity algorithm for drug repurposing in Alzheimer’s disease using automated machine learning

**DOI:** 10.1186/s13040-026-00550-4

**Published:** 2026-03-30

**Authors:** Sisi Shao, Pedro Henrique Ribeiro, Alena Orlenko, Katie M. Cardone, Christina M. Ramirez, Li Shen, Marylyn D. Ritchie, Jason H. Moore

**Affiliations:** 1 Department of Biostatistics, Fielding School of Public Health, University of California, Los Angeles, CA USA; 2 Department of Computational Biomedicine, Cedars-Sinai Medical Center, Los Angeles, CA USA; 3 Department of Genetics, University of Pennsylvania, Philadelphia, PA USA; 4 Institute for Biomedical Informatics, University of Pennsylvania, Philadelphia, PA USA; 5 Division of Informatics, Department of Biostatistics, Epidemiology and Informatics, University of Pennsylvania, Philadelphia, PA USA

**Keywords:** Alzheimer’s disease, Drug repurposing, Graph neural networks, Automated machine learning, MAP-elites, Novelty search

## Abstract

**Background:**

Alzheimer’s disease (AD) remains a major therapeutic challenge, characterized by high clinical trial failure rates and limited efficacy of current treatments. Drug repurposing offers a faster, lower-risk route to new therapies; however, existing computational approaches often prioritize predictive accuracy over mechanistic novelty and interpretability, both of which are critical for clinical translation.

**Results:**

We introduce a quality-diversity Automated Machine Learning (AutoML) framework that integrates biologically informed graph neural network (GNN) embeddings with a MAP-Elites-guided search to discover predictive yet mechanistically distinct therapeutic hypotheses. Drugs and genes are embedded using GraphSAGE and variational graph autoencoders trained on the Alzheimer’s Knowledge Base (AlzKB), with a clustering loss used to anchor known AD entities and define dimensions of biological novelty. In an AD case study using matched ADSP GWAS-derived features, our framework successfully recovered known drug–gene relationships and identified robust consensus candidates across independent validation runs. Most notably, the search consistently prioritized **Triclosan**—a recently identified environmental risk factor for AD neuroinflammation—and the **Ketamine/Quazepam** pair, suggesting a model-driven preference for restoring synaptic E/I balance. Furthermore, exploratory leads such as Exemestane and Felodipine were identified in underrepresented biological niches, supported by enrichment in oxidative stress and autophagy pathways. The framework demonstrated high stability across multiple random seeds and a **48% reduction in computational cost** compared to standard multi-objective evolutionary baselines.

**Conclusions:**

Beyond AD, this framework offers a generalizable strategy for integrating biomedical knowledge graphs with diversity-enhancing AutoML to accelerate the discovery of mechanistically novel drug candidates across complex polygenic diseases.

**Supplementary information:**

The online version contains supplementary material available at 10.1186/s13040-026-00550-4.

## Introduction

### Motivation for drug repurposing

Alzheimer’s disease (AD) remains a major therapeutic challenge, with current treatments like cholinesterase inhibitors and anti-amyloid monoclonal antibodies offering limited efficacy or carrying significant safety concerns [1, 2]. Given the high failure rate and cost of de novo drug development, drug repurposing—identifying new uses for existing drugs—offers a critical alternative pathway [3]. While computational strategies have nominated candidates such as bumetanide and sildenafil [4], systematically identifying mechanistically novel candidates remains a bottleneck. A comprehensive review of the current AD therapeutic landscape and the detailed rationale for repurposing is provided in Additional file 1 (Section S4).

### Role of machine learning in drug repurposing

The complexity of AD necessitates data-driven methods to identify novel therapeutic opportunities. Recent ML approaches—from GNNs to generative AI and multi-omics integration—have successfully nominated repurposing candidates by analyzing vast datasets [5–8]. Automated ML (AutoML) frameworks, such as the Tree-based Pipeline Optimization Tool (TPOT), further accelerate this process by automating model design [9, 10]. However, these tools often operate as “black boxes” that prioritize predictive accuracy over biological interpretability and the integration of domain knowledge. This is a critical limitation where a clear mechanistic rationale is paramount for clinical translation.

Our work builds on TPOT2, which enhances AutoML with a flexible, graph-based pipeline representation well-suited for multi-objective optimization [11]. This architecture allows for a more nuanced search that can be guided by curated domain knowledge to balance predictive performance with the discovery of interpretable and biologically plausible therapeutic strategies, directly addressing the key challenges in computational drug repurposing for AD.

### Key contributions and novelty

We introduce a novel AutoML-driven drug repurposing framework that uniquely synergizes graph-based feature representations, embedding-driven clustering, and a diversity-enhancing evolutionary search paradigm to overcome critical limitations in computational drug discovery for Alzheimer’s Disease. Our key contributions, which collectively advance the state-of-the-art, include:

- Biologically-Informed GNN Embeddings for Adaptive Feature Engineering: We develop curated domain-driven, adaptable feature representations by embedding drugs and genes based on their proximity to known AD-related entities within AlzKB. These GNN-derived embeddings critically leverage domain knowledge while offering crucial flexibility in dimensionality—a balance often missed by more rigid feature engineering techniques—allowing tailored analysis that scale with the available data.
- Clustering-Enhanced Feature Structuring for Improved Generalization: Our framework structures feature sets by clustering drugs based on their GNN embedding distances to known AD drugs. This innovative step not only improves model generalization by grouping mechanistically similar candidates but also provides deeper mechanistic insights by revealing higher-order relationships within the drug space.

A persistent challenge in ML-driven drug discovery is the inherent opacity (black-box nature) of complex models. Traditional ML often provides only *model-level explainability* (e.g., post-hoc feature importance like SHAP values for a single trained model), which fails to explain why certain biological pathways were selected over others during the search phase. Our framework directly confronts this challenge by offering *global search-landscape transparency*.

By leveraging graph-based feature representations derived from GNN embeddings and integrating MAP-Elites, as proposed by [12], we systematically partition the solution space into a grid. Each cell archives solutions based on both performance metrics (quality) and behaviorally distinct feature properties (our dimensions of biological novelty). The MAP-Elites grid visualization serves as a powerful macroscopic tool. Rather than acting as a black-box oracle, it illustrates exactly how distinct feature sets—representing different biological mechanisms—differ from each other and impact predictive performance. In the context of this study, this transparency in navigating the trade-offs between biological novelty and predictive quality is what we define as biological interpretability, ultimately guiding the identification of plausible drug-repurposing strategies that purely optimization-focused methods might fail to uncover.

## Methods

### Biologically-informed feature representation with GNNs

Graph Neural Networks (GNNs) are a class of machine learning models designed for graph-structured data, where the relationships between entities are as important as their individual attributes [13]. By iteratively propagating information across the graph’s connections, GNNs learn low-dimensional vector representations, or embeddings, for each node. These embeddings capture both the node’s intrinsic features and its broader structural context, making them exceptionally powerful for downstream machine learning tasks.

In this study, we developed two distinct GNN architectures to generate features from the AlzKB knowledge graph, a crucial design choice for modeling the unique characteristics of genes and drugs. For gene embeddings, we employed GraphSAGE [14], an inductive GNN whose neighborhood sampling mechanism is well-suited for capturing a gene’s local context. For drug embeddings, we used a Variational Graph Autoencoder (VGAE) [15], a probabilistic model that excels at representing the inherent uncertainty in drug-target interactions. This dual-GNN approach provided a robust foundation for feature engineering.

**Fig. 1:**
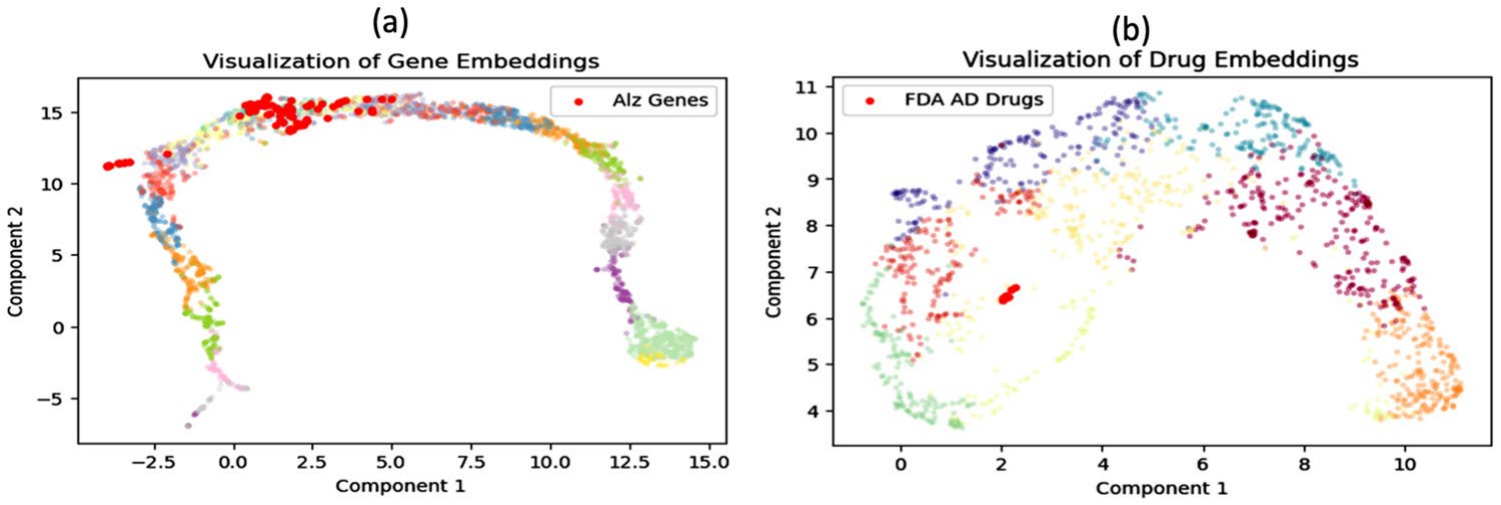
UMAP visualization of GNN-derived embeddings. (**a**) GraphSAGESAGE embedding of 3205 protein‑coding genes. The 101 known AD genes (red points) form a tight, cohesive cluster due to the domain-specific clustering loss. The remaining genes occupy distinct topological arms reflecting functional modules: the left arm is enriched for core amyloid pathology and neuroprotection (e.g., *APP*, *ADAM10*, *VEGFA*), while the right arm captures ion homeostasis and membrane excitability (e.g., *CACNA1H*, *KCNMB1*, *SLC22A2*). (**b**) VGAE embedding of 1411 drugs. The 11 FDA‑approved AD drugs (red points) cluster together, whereas the other 1400 drugs are distributed across multiple embedding neighborhoods. Colors in the background indicate K‑means clusters used for visualization

A key innovation in our work is the integration of a clustering loss objective, $\mathcal{L}_{\mathrm{cluster}}$, into the training of both GNNs. The primary motivation for this design stems from the necessity to infuse biological coherence and interpretability directly into the learned embeddings. While standard GNNs excel at capturing topological relationships, they may not inherently group entities based on higher-order functional similarities crucial in biology. To explicitly address this, $\mathcal{L}_{\mathrm{cluster}}$ was designed to enforce intra-class compactness by minimizing the maximum pairwise Euclidean distance within predefined sets of known AD-related entities. Specifically, this loss function encourages a compact clustering of 101 known AD-related genes in the gene embedding space, and similarly for 11 known AD drugs in the drug embedding space. We acknowledge that more sophisticated objectives, such as contrastive learning methods that explicitly maximize inter-class distances, could potentially yield more discriminative embeddings. However, our choice of a more direct compactness objective serves as a deliberate and computationally efficient inductive bias. The primary goal of this work is not to achieve perfect class separation across the entire embedding space, but rather to ensure that the embeddings of known, functionally related entities are sufficiently coherent to serve as a reliable anchor for defining biological novelty. By imposing this strong biological prior—that entities known to be associated with a disease should share a cohesive latent signature—we effectively guide the embedding process towards representations that are both topologically informed and functionally relevant for the downstream task of novelty-guided search. This approach proved sufficient for our goals, as evidenced by the clear separation of the target clusters in the embedding space (Fig. 1a–b). To further validate this design, we performed a post-hoc topological analysis of the embedding functional modules and an ablation study demonstrating that removing $\mathcal{L}_{\mathrm{cluster}}$ leads to a loss of biological coherence among AD entities (see Additional file 1: Section S1.3 and Figure S3). Additionally, the clinical relevance of the learned drug embeddings was validated by comparing latent distances against the Anatomical Therapeutic Chemical (ATC) classification system, confirming that pharmacologically related drugs exhibit significantly higher proximity (see Additional file 1: Figure S2).

#### Connection to positive-unlabeled (PU) learning

Methodologically, our embedding strategy conceptually parallels PU Learning [16]. In drug repurposing, ground-truth labels exhibit a fundamental asymmetry: while we possess a set of known AD-associated drugs (Positives), the remaining entities cannot be reliably classified as non-therapeutic (Negatives), but rather as “Unlabeled” candidates that may contain undiscovered treatments. Standard supervised approaches that treat unlabeled data as negatives risk penalizing novel discoveries. Instead of training a traditional binary classifier to separate positives from inferred negatives, our framework utilizes the clustering loss ($\mathcal{L}_{\mathrm{cluster}}$) to function as a lightweight, representation-level analogue of PU learning. By enforcing intra-class compactness exclusively for the Positive class (known AD entities), we learn a latent data description where the Positive class forms a coherent core. The “novelty” of a candidate is then rigorously defined not as the probability of being negative, but as its geometric distance from this anchored positive cluster in the embedding space. This allows MAP-Elites to explore the Unlabeled space systematically, prioritizing candidates that are mechanistically distinct (distant) yet functionally relevant.

Regarding the weight of the clustering loss term, $\lambda_{\mathrm{cluster}}$, our framework incorporates $\mathcal{L}_{\mathrm{cluster}}$ with a unit weight (i.e., $\lambda_{\mathrm{cluster}} = 1$). This design choice is twofold: theoretically, it reflects our inductive bias that biological coherence is as fundamental as graph topology; empirically, we observed that the reconstruction and clustering losses operate within comparable numerical magnitudes, ensuring balanced gradient contributions from both objectives. This alignment allowed for effective embedding learning without the need for extensive hyperparameter grid searching. For other standard regularization terms, such as L2 regularization, a conventional weight of $10^{-2}$ was applied to prevent overfitting.

The full architectural details, mathematical formulations, and implementation specifics for both GNN models are provided in the Additional file 1 (Section S1.1).

While AlzKB integrates diverse evidence streams—including clinical trials, text-mined associations, and GWAS results with varying confidence levels—our framework treats this database as a structured biological prior to anchor the novelty search, rather than as an absolute ground truth for therapeutic efficacy.

### Feature set construction using GNN-derived embeddings

**Fig 2:**
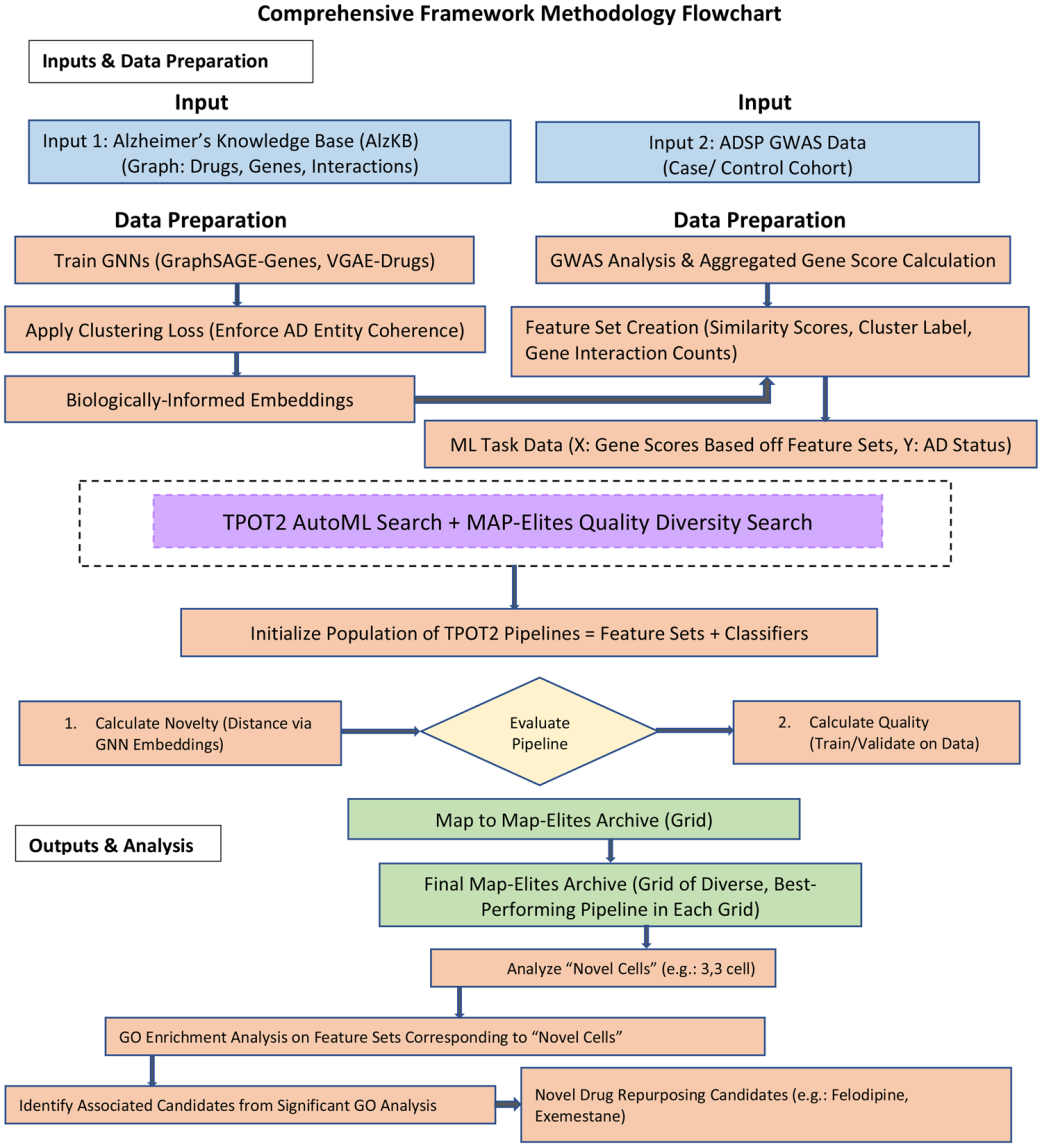
Comprehensive framework of the proposed novelty-guided drug repurposing methodology. The pipeline integrates two main data inputs: the Alzheimer’s knowledge Base (AlzKB) for graph-based features and ADSP GWAS data for genetic scores. GNNs are trained on AlzKB data to generate embeddings with a clustering loss to enforce biological coherence. GWAS data undergoes analysis for aggregated gene score calculation, which, along with the GNN embeddings, forms the input for the TPOT2+MAP-Elites AutoML search. This search optimizes for both predictive quality and mechanistic novelty, generating diverse, high-quality pipelines. The final output identifies novel drug candidates for repurposing based on their associated gene sets and their performance in the optimized pipelines

Building on our GNN embeddings, we construct the final feature set for TPOT2 through a multi-step process, summarized in Fig. 2. Our primary goal was to create robust, biologically-grounded features that avoid the inefficiencies caused by feature sparsity, a common issue where many drugs are linked to only a few known gene targets.

To achieve this, our strategy leverages the dense, low-dimensional GNN embeddings as a basis for feature creation. We first quantified drug similarity by computing three complementary distance metrics (Canberra, Euclidean, and Cosine) between the drug embeddings. We then employed K-Means clustering to group drugs based on these continuous, embedding-based distance features. To ensure the robustness of this parameter choice, the optimal number of clusters ($k= 24$) was rigorously determined via the Elbow Method applied to the within-cluster sum of squares (see Additional file 1: Figure S1).

A crucial preprocessing step was implemented to mitigate potential bias from high-degree “hub” drugs (polypharmacology). An analysis of the drug-gene interaction distribution in AlzKB revealed a heavy-tailed distribution with a median degree of 10 and a mean of 36.33, indicating that the network average is skewed by a subset of highly promiscuous compounds. These broad-spectrum agents can disproportionately influence K-Means centroids, masking the specific mechanistic signals of more targeted therapies.

To address this, we adopted a specificity-focused filtering threshold of 20 gene interactions. This threshold, representing approximately twice the median degree, effectively separates the highly promiscuous agents (top $\sim$28%) from the majority of the pharmacopeia ($\sim$72%) that exhibits more defined target profiles. By isolating drugs exceeding this threshold into a dedicated “hub” group (labeled as −1), we preserve the structural integrity and biological interpretability of the primary clusters ($k=24$) while preventing non-specific connectivity from dominating the embedding space. Importantly, these high-degree drugs were retained in the final feature set to preserve their broad interaction data, ensuring they contribute to the model without distorting the centroids of specific mechanistic clusters.

The final feature set integrates these GNN-based similarity distances, cluster labels, and raw gene interaction counts. We explicitly included raw gene interaction counts—specifically defined here as the total number of a drug’s gene targets within the AlzKB knowledge graph, rather than its global degree centrality (which would inappropriately include links to diseases or other drug classes)—to allow the AutoML model to empirically determine whether broad-spectrum target engagement or high specificity is more predictive for AD therapeutic efficacy. Full details on the metrics and clustering are provided in Additional file 1 (Section S1.2).

### TPOT2 with MAP-Elites for AutoML-driven drug classification

**Fig. 3:**
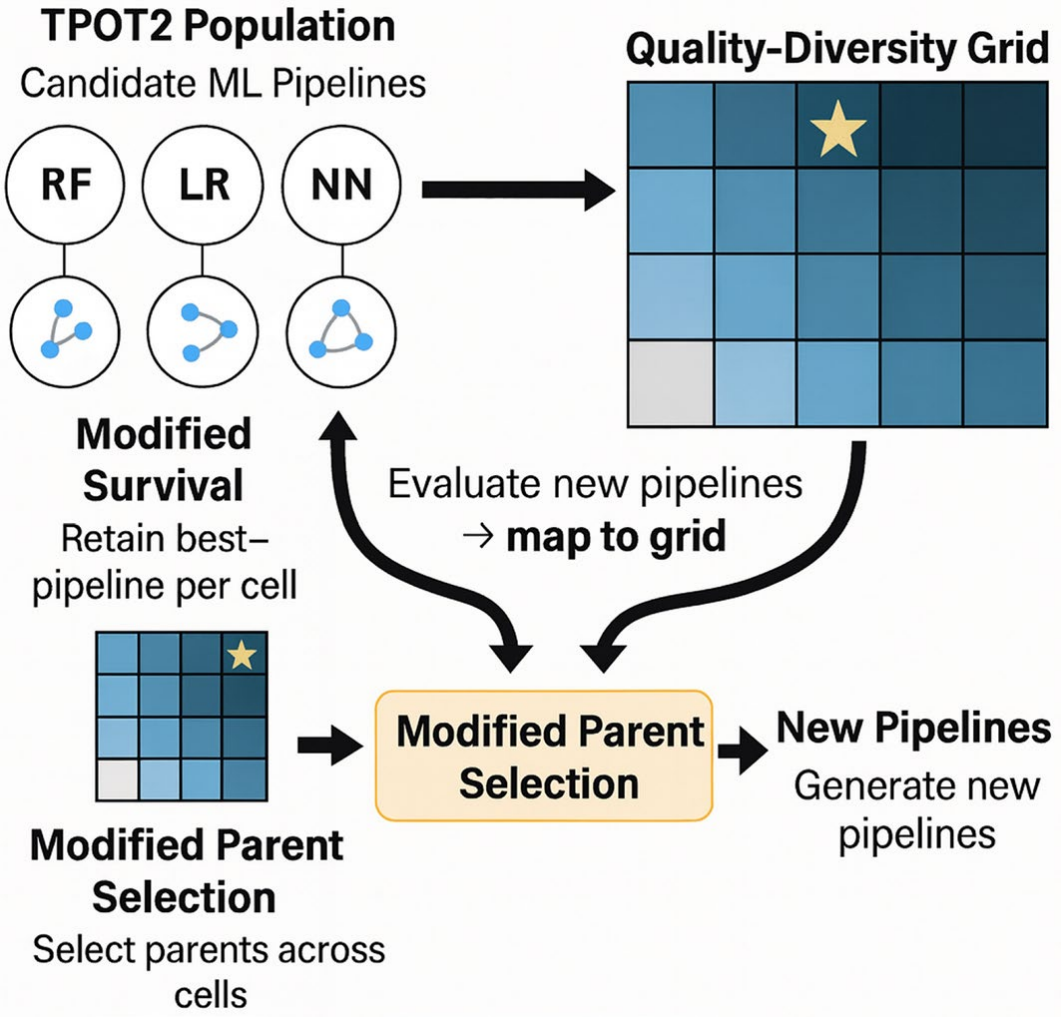
Conceptual integration of MAP‑Elites into TPOT2: we start with the TPOT2 population, partition the search space into a quality‑diversity grid, apply modified survival and parent selection, and generate new offspring that are mapped back into the archive

We developed a novel AutoML framework by integrating the quality-diversity algorithm MAP-Elites into TPOT2 to overcome the limitations of standard ML approaches in drug discovery. Standard methods often prioritize pure predictive accuracy, leading to “black box” models that tend to converge on well-characterized but biologically redundant hypotheses, potentially mimicking historical failures rather than identifying new therapeutic avenues. In contrast, our framework operationalizes *novelty search* by utilizing GNN-derived embedding distances to guide the evolutionary algorithm. This compels the search to actively explore diverse and biologically distinct regions of the feature space, identifying robust candidates in under-explored mechanisms (Fig. 3).

#### Cohort construction and population stratification control

We utilized a case-control cohort from the Alzheimer’s Disease Sequencing Project (ADSP), which aggregates whole-genome sequencing data across diverse ancestries [17]. To prevent spurious associations arising from ancestral heterogeneity, we adopted a rigorous matching-based correction approach following the framework proposed by Orlenko et al. [18].

##### Quality control and extraction of independent loci

To ensure that principal components (PCs) accurately capture large-scale ancestry differences rather than local linkage signals, we first selected a subset of independent SNP loci through standard genetic quality control (QC) filters [19, 20]:

- **Minor Allele Frequency (MAF):** Rare variants (MAF $$\leq 0.02$$) are prone to genotyping errors and contribute little to global ancestry variance. We retained only variants with MAF $$ > 0.02$$ to stabilize the PCA covariance structure [21].
- **Hardy–Weinberg Equilibrium (HWE):** Variants deviating from expected proportions (exact test $p \leq 1\times10^{-7}$) were excluded to remove technical artifacts or loci under strong selection [22].
- **Linkage Disequilibrium (LD) Pruning:** To prevent correlated SNP clusters from dominating the PCA, we applied LD pruning (window = 100, step = 10, $R^2 < 0.1$), retaining only one representative SNP per LD block [23].

Using these independent loci, we performed Principal Component Analysis (PCA) to extract the top eight PCs (PC1–PC8) summarizing ancestral variation.

##### Propensity score estimation and matching

Instead of using PCs directly as covariates, we reduced the high-dimensional ancestry information into a single scalar metric: the propensity score. A logistic regression model was fitted using AD status as the outcome:

1 $$\mathrm{logit}(\pi_i) = \beta_0 + \beta_1 PC1_i + \cdots + \beta_8 PC8_i$$

where $\pi_i$ denotes the predicted probability that individual $i$ belongs to the AD case group given their ancestry profile. We then applied one-to-one nearest-neighbor matching on these scores using the psmpy package [24]. The final analytic dataset comprised 22,560 individuals (11,280 matched case-control pairs). The cohort was carefully balanced across the major ancestral groupings represented in the ADSP—including Non-Hispanic White, Black/African American, and Hispanic/Latino populations—to provide a genetically homogeneous foundation for downstream analysis.

#### Optimization-driven conditional data partitioning

To ensure robust model generalization, we implemented a conditional data partitioning strategy using the Optuna hyperparameter optimization framework [25]. Unlike random splitting, this procedure ensured that the training, validation, and testing subsets (1:1:1 split) preserved two critical conditions: (1) the integrity of matched case/control pairs, and (2) comparable distributions of significant AD-associated SNPs.

##### Reference GWAS SNPs

We selected 30 representative AD-associated SNPs derived from a recent large-scale meta-analysis [26]. To ensure consistency with our cohort, we filtered the original list of 101 significant loci ($$p < 5\times10^{-8}$$) using strict QC criteria (MAF $$ > 0.1$$, HWE $$p > 1\times10^{-7}$$, LD $$R^2 < 0.8$$). The retained 30 SNPs served as markers to benchmark the distributional balance of our data splits.

##### Tree-structured parzen estimator (TPE) optimization

We treated the random seed determining the data partition as a hyperparameter to be optimized over 1000 trials. Optuna utilizes the Tree-structured Parzen Estimator (TPE) algorithm [27] to navigate this search space. TPE models two probability densities for the objective function value $f$:

2 $$l(x) = p(x \mid f < f^*), \quad g(x) = p(x \mid f \ge f^*)$$

where $f^*$ is a quantile threshold separating “good” from “bad” configurations. New split configurations $x_{\mathrm{new}}$ are sampled to maximize the expected improvement ratio:

3 $$x_{\mathrm{new}} = \arg\max_x \frac{l(x)}{g(x)}$$

This Bayesian approach allows the algorithm to focus its search on partition seeds that are most likely to satisfy our biological constraints. For each trial, we jointly optimized two objectives:

$$\begin{aligned}\max f_1 &= \mathrm{median}(M_{\mathrm{train}}, M_{\mathrm{val}}, M_{\mathrm{test}}), \\\min f_2 &= |M_{\mathrm{train}} - M_{\mathrm{val}}| + |M_{\mathrm{train}} - M_{\mathrm{test}}| + |M_{\mathrm{val}} - M_{\mathrm{test}}|.\end{aligned}$$

where $M_k$ denotes the median $-\log(p)$ of the 30 representative SNPs in subset $k$. The final selected split maximized signal strength ($f_1$) while minimizing distributional divergence ($f_2$).

#### Feature engineering and evaluation

##### Aggregated gene scores

To translate genomic data into interpretable predictors, we computed gene-level scores for variants with GWAS $p < 0.05$. For each individual, the score for a specific gene was calculated as:

4 $$\text{Gene Score} = \frac{\sum (\beta \times \mathrm{Genotype})}{n_{\mathrm{variants}}}$$

where $\beta$ is the GWAS log odds ratio, *Genotype* is the allele dosage (0, 1, or 2), and $n_{\mathrm{variants}}$ is the mapped variant count.

These scores were then aggregated within the biologically informed feature sets defined by our GNN-guided clustering. For an individual $i$ and feature set $\mathcal{F}$:

5 $$S_{\mathcal{F},i} = \sum_{g \in \mathcal{F}} \mathrm{GeneScore}_{i,g}$$

This aggregation reduces the high-dimensional gene space to interpretable modules while preserving quantitative signal strength.

##### Pipeline evaluation

**Fig. 4:**
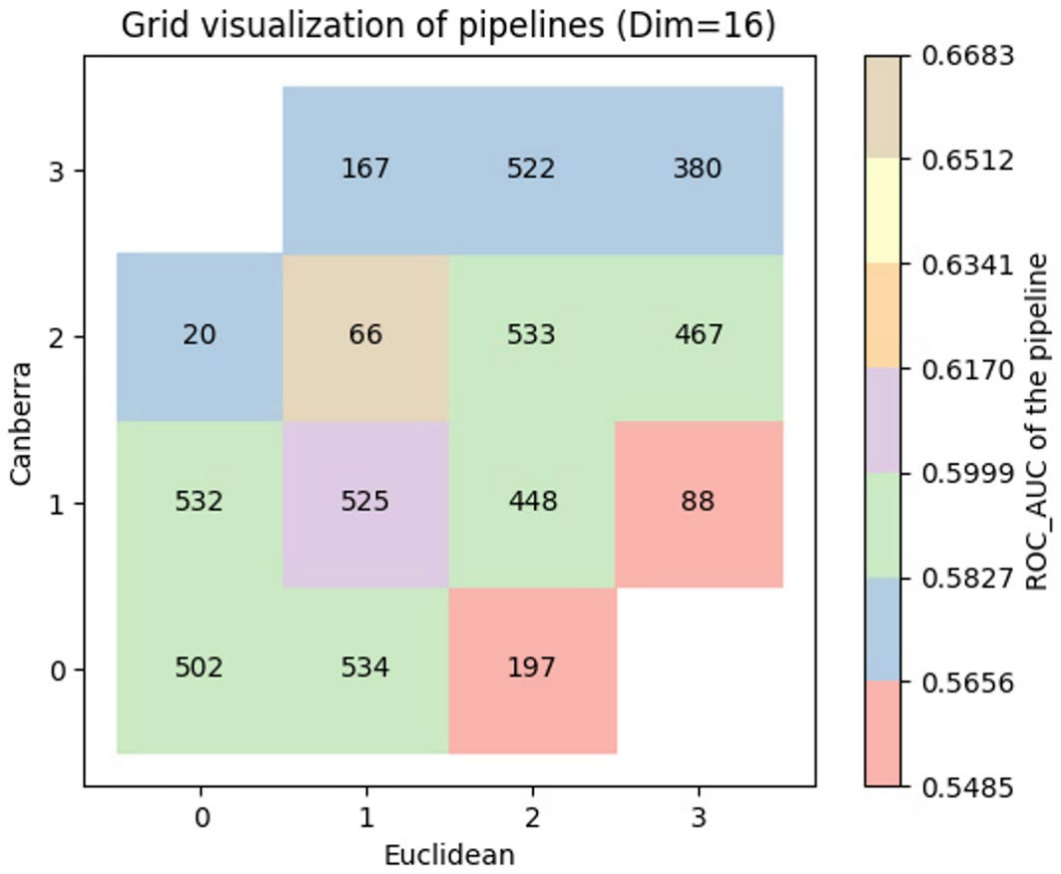
MAP-Elites grid visualization of discovered ML pipelines. The illustrative grid represents one generation of MAP-Elites search using a GNN embedding dimension of 16. Cells are arranged by behavioral descriptors (quartiles of Euclidean and Canberra distances), with color encoding the pipeline’s ROC-AUC score and numbers denoting the specific feature set utilized

Each aggregated feature set score $S_{\mathcal{F},i}$ served as the input predictor for the TPOT2 pipelines. We employed a logistic regression model to classify AD status, using the Area Under the Receiver Operating Characteristic Curve (AUC-ROC) to quantify performance. This metric guided the evolutionary selection process within the MAP-Elites grid (Fig. 4). To rigorously assess the algorithmic stability and performance differences across multiple random initializations, we employed variance equality testing (F-test) to compare the standard deviations of the retrieval metrics, alongside Welch’s t-tests to compare mean performance, ensuring robust statistical validation of our comparative baselines.

##### Interpreting the MAP-elites grid for candidate prioritization

To clarify the added value of the MAP-Elites visualization, it is essential to understand how the feature sets in each grid cell differ and how they guide candidate prioritization. Each cell in the grid represents a distinct biological niche defined by our GNN-derived novelty metrics (e.g., Euclidean and Canberra distances from known AD drugs). Feature sets mapped to cells near the origin (e.g., 0,0) represent drug-gene networks structurally and functionally highly similar to existing FDA-approved AD therapies (low novelty). Conversely, cells in the outer boundaries (e.g., 3,3) contain gene sets functionally distinct from current therapies.

This search-landscape transparency provides a clear, actionable guide for drug prioritization based on specific clinical use cases. For example, if researchers aim to identify an adjunctive therapy with a non-overlapping mechanism to current standard-of-care (e.g., targeting neuroinflammation rather than the cholinergic system), they can specifically prioritize high-performing drug candidates extracted from the high-novelty quadrants. Conversely, if the goal is to discover a “me-too” drug-one that shares the established mechanism of an existing AD drug but possesses a potentially superior systemic safety profile or blood-brain barrier penetrance—researchers would query the high-performing pipelines within the low-novelty cells. By explicitly linking grid position to mechanistic novelty, the framework transforms abstract feature diversity into a practical prioritization tool.

##### Clinical evaluation metrics

To evaluate the pipelines within the specific context of clinical AD classification, we contextualize our metrics using the ADSP cohort. Let $AD_{Total}$ be the total true AD patients and $HC_{Total}$ be the true healthy controls. Based on the model’s predictions using a drug’s specific feature set, let $AD_{detected}$ denote correctly identified patients, $AD_{missed}$ denote AD patients incorrectly classified as healthy, and $HC_{flagged}$ denote healthy controls falsely classified as having AD.

We define our key retrieval metrics as follows:

- **Recall (Sensitivity):** $\frac{AD_{detected}}{AD_{Total}}$. A high Recall ensures the candidate drug’s target network is highly penetrant and reflects a shared disease mechanism across the majority of AD patients.
- **Precision:** $\frac{AD_{detected}}{AD_{detected} + HC_{flagged}}$. This minimizes the risk of prioritizing drugs that target non-AD-specific pathways (e.g., normal aging), which could lead to off-target effects.
- **F1-Score:** The harmonic mean of Precision and Recall, providing a balanced evaluation against the imbalanced nature of clinical cohorts.
- **ROC-AUC:** Mathematically representing $P(Score(AD_i) > Score(HC_j))$, a high AUC indicates the drug’s target network exhibits a globally different activation state in AD patients compared to healthy controls.
- **Recall SD:** For $N$ independent runs, $SD_{Recall} = \sqrt{\frac{1}{N-1} \sum_{k=1}^{N} (Recall_k - \overline{Recall})^2}$. Maintaining a low Recall SD ensures the prioritized drugs represent universally robust mechanisms resilient to initialization noise.

#### Search strategy: MAP-elites vs. random search

To validate the utility of our quality-diversity approach, we explicitly compare it against a baseline Random Search strategy.

- **Random Search:** Evaluates a large single generation (10,000 individuals) without optimizing diversity, representing a pure exploration baseline without memory.
- **MAP-Elites:** Maintains an archive of diverse models, evolving over 100 generations to ensure both high performance and feature diversity, actively illuminating the search space defined by our GNN embeddings.

## Results and discussion

### Exploration efficacy: MAP-Elites vs. random search

**Fig. 5:**
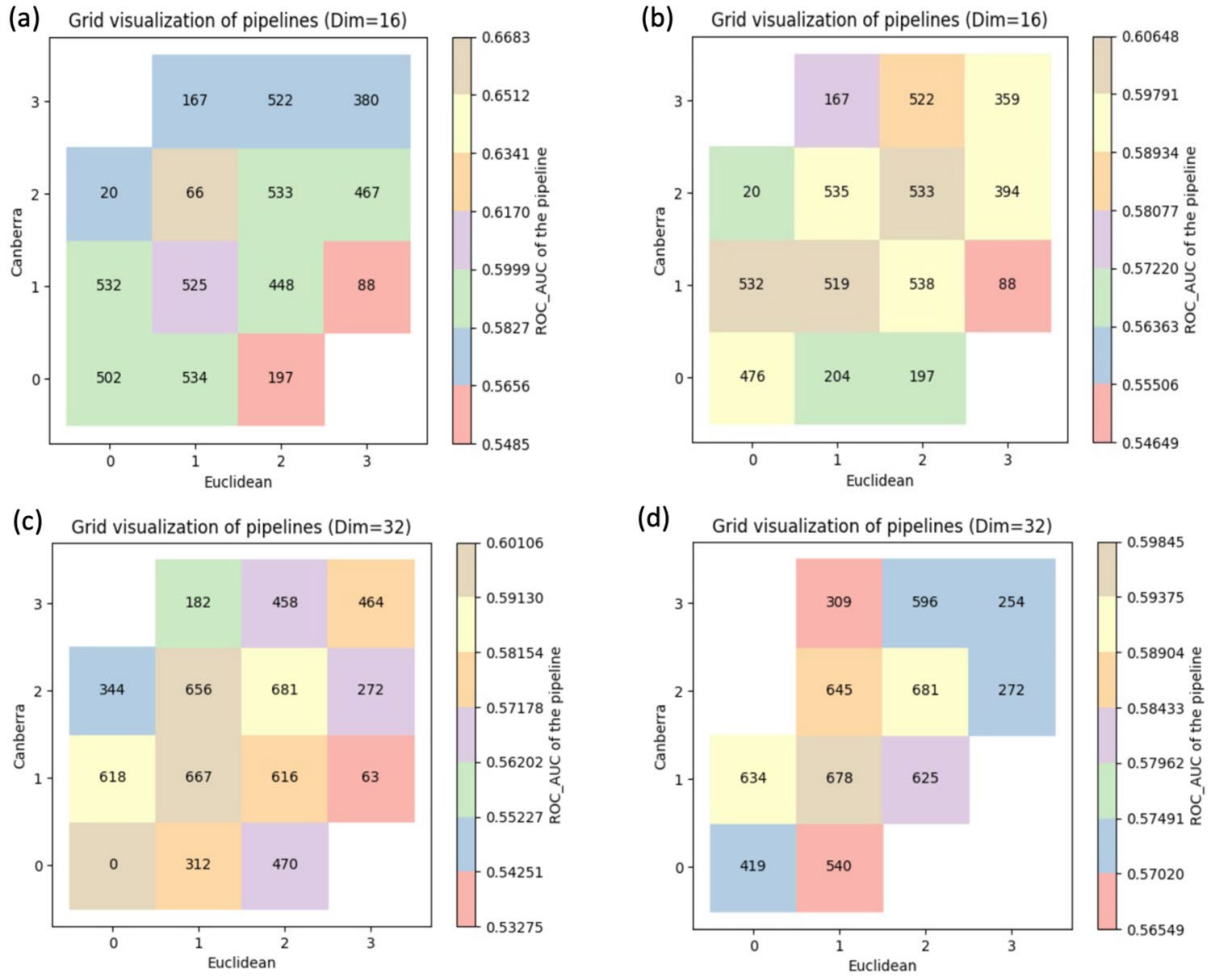
Comparison of evolved pipelines across the behavior space. While random search (**a, c**) manages to sample high-novelty regions, it suffers from a significant degradation in AUC as novelty increases. In contrast, MAP-Elites (**b, d**) effectively discovers high-quality solutions without sacrificing performance. The observed “performance plateau” in MAP-Elites refers to its ability to sustain a high-quality AUC score across the entire breadth of the novelty space (e.g., achieving comparable performance at both the low-novelty cell (0,0) and the high-novelty cell (3,3))

Empirical analysis of the search coverage demonstrates that Random Search fails to effectively navigate the high-dimensional biological space. Table 1 shows that MAP-Elites consistently achieves significantly greater average distances from known AD genes (e.g., +105.9% Euclidean distance in Dim 32). The performance and coverage trade-offs of these two approaches are visually compared in Fig. 5, illustrating the “performance plateau” achieved by MAP-Elites. In this context, the performance plateau refers to the algorithm’s ability to maintain consistently high predictive accuracy (AUC) across a wide range of biologically diverse and highly novel feature spaces, avoiding the severe performance degradation typical of unguided exploration. Importantly, this observation is supported by an empirical limitation of the Random Search baseline: in the Dim = 32 setting, Random Search selected gene sets that were insufficient in size and coherence to support downstream GO enrichment analysis.

**Table 1:** Comparison of MAP-Elites vs. Random search. MAP-Elites achieves significantly greater average distances from known AD genes, indicating broader exploration

Embedding Dimension	Cosine Distance (%)	Canberra Distance (%)	Euclidean Distance (%)
16	+49.7%	+20.7%	+24.4%
32	+310.5%	+87.2%	+105.9%

### Methodological proof-of-concept: comparative benchmarking

To address the need for rigorous methodological validation and demonstrate that this framework constitutes a robust proof-of-concept, we performed a comprehensive benchmarking against state-of-the-art baselines: **XGBoost** (representing pure exploitation/standard ML) and **NSGA-II** (representing standard multi-objective evolution).

As shown in Table 2, while XGBoost achieves marginally higher raw AUC, it exhibits extreme instability in candidate retrieval (Recall SD $\pm$ 0.098). To rigorously validate these observations, we performed variance equality testing (F-test) to evaluate algorithmic stability and Welch’s t-tests to compare mean performance across the independent runs. The complete statistical results are summarized in Table 3.

**Table 2:** Comparative benchmarking of predictive performance and stability (mean $\pm$ SD across 5 seeds). MAP-Elites demonstrates superior stability in high-novelty regions compared to baselines

Feature Dim	Method	ROC-AUC	PR-AUC	F1-Score	Precision	Recall	Recall SD
*Dim 16 (Primary Case)*							
	**XGBoost**	0.610 $\pm$ 0.002	0.575 $\pm$ 0.007	0.603 $\pm$ 0.035	0.570 $\pm$ 0.005	**0.648**	$\pm$ 0.098
	MAP-Elites (Novel)	0.591 $\pm$ 0.002	0.560 $\pm$ 0.002	0.598 $\pm$ 0.008	0.556 $\pm$ 0.001	**0.648**	$\pm$ **0.017**
	NSGA-II (Novel)	0.580 $\pm$ 0.005	0.553 $\pm$ 0.004	0.588 $\pm$ 0.014	0.549 $\pm$ 0.004	0.633	$\pm$ 0.032
*Dim 32 (Exploration Case)*							
	MAP-Elites (Novel)	**0.567** $\pm$ **0.002**	0.543 $\pm$ 0.003	0.561 $\pm$ 0.012	0.542 $\pm$ 0.004	0.581	$\pm$ **0.022**
	NSGA-II (Novel)	0.561 $\pm$ 0.008	0.539 $\pm$ 0.006	0.535 $\pm$ 0.046	0.543 $\pm$ 0.005	0.535	$\pm$ 0.083

**Table 3:** Statistical validation of algorithmic stability (MAP-Elites vs. Baselines)

Comparison (Baseline)	Feature Space	Mean Diff. (Welch’s T-Test)	Variance Diff. (F-Test)	Conclusion
MAP-Elites vs. XGBoost	Dim 16 vs. All Features	$p = 0.99$(ns)	$p < 0.01$(**)	MAP-Elites significantly reduces variance.
MAP-Elites vs. XGBoost	Dim 32 vs. All Features	$p = 0.25$(ns)	$p < 0.01$(**)	Stability superiority is maintained in high dimensions.
MAP-Elites vs. NSGA-II	Dim 16 (Primary)	$p = 0.45$(ns)	$p = 0.24$(ns)	Comparable stability in lower dimensions.
MAP-Elites vs. NSGA-II	Dim 32 (Exploration)	$p = 0.28$(ns)	$p = 0.024$(*)	MAP-Elites is significantly more stable.

*Notes: ns = not significant (*$p > 0.05$); * = significant ($p < 0.05$); ** = highly significant ($p < 0.01$).

The statistical analysis confirmed that the retrieval variability in the full-feature XGBoost baseline is significantly higher than the highly stable performance of MAP-Elites in the primary setting (Recall SD $\pm$ 0.017), yielding a highly significant difference in stability ($p < 0.01$, F-test). Concurrently, Welch’s t-test confirmed no significant difference in their mean Recall ($p = 0.99$), demonstrating that MAP-Elites successfully mitigates random initialization variance without sacrificing predictive power. Crucially, this stability superiority over the global XGBoost baseline is strictly maintained even when MAP-Elites navigates the highly complex 32-dimensional exploration space ($p < 0.01$, F-test; with comparable mean Recall, $p = 0.25$). Furthermore, within this high-dimensional setting (Dim 32), MAP-Elites significantly outperforms standard multi-objective evolution (NSGA-II) in stability ($p = 0.024 < 0.05$, F-test). This benchmarking rigorously confirms that our quality-diversity approach balances exploration and exploitation more effectively than standard baselines across varying degrees of complexity.

The trade-off between global ROC-AUC and Recall stability stems from how these algorithms navigate complex biological data. Traditional baselines like XGBoost greedily optimize for global accuracy. In high-dimensional genomic spaces, this approach often causes search instability, as the model struggles to differentiate between redundant signals and becomes highly sensitive to initialization noise. In contrast, MAP-Elites avoids this greedy convergence. By explicitly selecting the structurally most novel candidates—those at the geometric boundaries of the GNN embedding space—the algorithm trades a marginal drop in global AUC for a robust performance plateau. This Quality-Diversity approach ensures stable, reproducible retrieval of disease signals. For clinical drug repurposing, maintaining a consistently high Recall with minimal variance is critical; it indicates that a candidate drug targets a shared, penetrant disease mechanism, ultimately minimizing missed therapeutic opportunities.

Crucially, this greedy optimization directly impacts candidate discovery. Empirical analysis revealed that XGBoost, optimizing solely for global accuracy on the full feature space, completely failed to prioritize the specific gene targets associated with our highly novel consensus candidates, such as Triclosan and the Ketamine/Quazepam pair. Similarly, the standard multi-objective baseline (NSGA-II) exhibited severe directional loss, failing to reliably recover these targets across independent runs (detailed in Additional file 1: Sections S2.5 and S2.6). This confirms that the integrated MAP-Elites framework is uniquely responsible for illuminating these mechanistically distinct therapeutic hypotheses.

### Gene ontology (GO) analysis: mechanistic coherence in exploratory runs

To verify that the novel feature sets represent coherent biological modules, we conducted a GO analysis using the Harmonizome tool [28]. We visualized networks for representative pipelines from our initial exploratory analysis. The complete sets of genes prioritized in these exploratory runs, which formed the basis for the GO enrichment, are provided in Additional file 1: Table S1.

#### Findings from exploratory GO analysis

- **Dim = 16 (Exemestane Case):** As visualized in Additional file 1: Figure S6, the network revealed functional modules enriched for steroid catabolic processes (Module M1), biologically aligning with Exemestane’s mechanism as an aromatase inhibitor.
- **Dim = 32 (Felodipine Case):** The analysis revealed a clustering of genes involved in ion channel regulation (e.g., *GABRA1*) and drug metabolism (Additional file 1: Figure S7), reflecting the biological context of calcium channel blockers.

### Uncovering robust therapeutic hypotheses

Following the exploratory phase, we focused on identifying candidates that repeatedly appeared across the **5 independent validation seeds**.

#### Robust consensus candidates: Triclosan and Ketamine

The algorithm consistently converged on specific candidates, validating their significance (Table 4; robustness visualized in Additional file 1: Figures. S4–S5).

- **Triclosan (Risk Factor Identification):** In Dim = 16, Triclosan was retrieved in 4 out of 5 runs. While historically an antimicrobial, recent 2025 studies have identified it as a novel environmental risk factor for AD, inducing neuroinflammation via the TNF-$\alpha$ axis [29, 30].
- **Ketamine and Quazepam (Therapeutic E/I Balance):** In Dim = 32, the search recovered **Ketamine** (NMDA antagonist) in 2 out of 5 runs. Notably, in specific seeds, Ketamine was co-retrieved with **Quazepam** (GABA agonist). As detailed in Additional file 1: Section S2.4, this co-occurrence suggests a model preference for restoring the **Excitation/Inhibition (E/I) balance**. While Ketamine modulates glutamatergic transmission [31], Quazepam enhances GABAergic inhibition [32], potentially offering a synergistic approach to reduce neuronal hyperexcitability.

**Table 4:** Mechanistic enrichment analysis of robust consensus candidates. Gene sets associated with Triclosan and Ketamine in high-performing pipelines were analyzed for functional enrichment

Candidate	Key Genes	Top Enriched GO Processes	Relevance to AD
**Triclosan**	*FASN, ACHE,*	$\bullet$ Fatty Acid Metabolic Process	Lipid dysregulation
	*PPARG, TNF*	$\bullet$ Regulation of Inflammatory Response	Neuroinflammation
		$\bullet$ Acetylcholine Catabolic Process	Cholinergic transmission
**Ketamine**	*GRIN2A, GRIN2B,*	$\bullet$ Glutamatergic Synaptic Transmission	Excitotoxicity regulation
	*BDNF, DLG4*	$\bullet$ NMDA Glutamate Receptor Activity	**Mechanism of Memantine**
		$\bullet$ Regulation of Membrane Potential	Synaptic plasticity

### Computational efficiency and study limitations

Regarding computational feasibility, benchmarking confirms that our novelty-driven approach is highly scalable. As detailed in Additional file 1 (Table S2), MAP-Elites demonstrated superior efficiency, particularly in high-dimensional search spaces. While the NSGA-II baseline slowed down significantly in the 32-dimensional setting (avg. 6.20 h), MAP-Elites maintained a low runtime (avg. 3.20 h), achieving a $\sim$48% reduction in computational cost.

**Limitations:** While our identified candidates—ranging from potential environmental risk factors like Triclosan to therapeutic agents like Ketamine, Quazepam, Exemestane, and Felodipine—are supported by recent independent studies reported in the literature, they remain in silico predictions requiring experimental validation. Additionally, regarding our methodological design: the outlier filtering threshold ($k=20$) was derived directly from the data distribution to mitigate hub bias (see Additional file 1: Section S1.2), and the clustering loss weight ($\lambda=1$) was grounded in the empirical observation of comparable loss magnitudes. However, we acknowledge that a comprehensive sensitivity analysis of $\lambda$ could further fine-tune the embedding space. Finally, we recognize that knowledge graphs like AlzKB are subject to ascertainment bias, where well-studied drugs with more recorded interactions may naturally cluster together; extending this framework to diverse, multi-omics datasets and utilizing specificity-focused filtering (as performed with our hub-drug removal) are critical steps to mitigate such biases.

## Conclusion and future work

This study addresses a critical challenge in computational drug repurposing for Alzheimer’s Disease: the need for methods that can systematically discover mechanistically novel and interpretable therapeutic hypotheses. We presented a framework that achieves this by uniquely integrating biologically-informed GNN embeddings with a MAP-Elites quality-diversity search within the TPOT2 AutoML pipeline. Our primary contribution is the use of a biomedical knowledge graph (AlzKB) to guide the evolutionary search, defining novelty based on an embedding’s distance to known AD-related entities. The identification of diverse candidates such as Exemestane and Felodipine from underrepresented regions of the feature space provides supporting evidence for the feasibility of this novelty-guided approach as a hypothesis-generation framework. By fusing graph-based biological priors with a diversity-aware evolutionary search, our work presents a methodological proof-of-concept for integrating biological knowledge into AutoML-driven drug repurposing. Future work will focus on enriching the biological data foundation by incorporating multi-omics datasets and exploring more advanced GNN architectures. We will also investigate more dynamic AutoML search paradigms using reinforcement learning and the integration of Large Language Models (LLMs) to automatically update the knowledge base. Ultimately, the most critical next step is the experimental validation of our predicted drug candidates through in vitro and in vivo studies, and the application of this framework to other complex neurodegenerative diseases, such as Parkinson’s Disease, Amyotrophic Lateral Sclerosis (ALS), and Huntington’s Disease.

## Electronic supplementary material

Below is the link to the electronic supplementary material.

Supplementary Material 1

## Data Availability

The genomic datasets supporting the conclusions of this article are available in the NIAGADS repository under controlled access (Alzheimer's Disease Sequencing Project; NG00067, Version 18; http://doi.org/10.60859/Z6Z9-9692). All source code, pre-trained embeddings, and processed intermediate files required to reproduce the findings of this study are available in the GitHub repository: https://github.com/SisiShao/Alzheimer-Drug-Repurposing-MAP-Elites.

## Abbreviations

AD: Alzheimer’s Disease
ADSP: Alzheimer’s Disease Sequencing Project
AlzKB: Alzheimer’s Knowledge Base
AutoML: Automated Machine Learning
CCB: Calcium Channel Blocker
GCN: Graph Convolutional Network
GNN: Graph Neural Network
GO: Gene Ontology
GWAS: Genome-Wide Association Study
MAP-Elites: Multi-dimensional Archive of Phenotypic Elites
ML: Machine Learning
NSGA-II: Non-dominated Sorting Genetic Algorithm II
PCA: Principal Component Analysis
PSM: Propensity Score Matching
ROC-AUC: Area Under the Receiver Operating Characteristic Curve
SNP: Single Nucleotide Polymorphism
TPOT: Tree-based Pipeline Optimization Tool
VGAE: Variational Graph Autoencoder
XGBoost: Extreme Gradient Boosting
