# Supplementary material for: A biology-based quality-diversity algorithm for drug repurposing in Alzheimer’s disease using automated machine learning
Source: BioData Min. 2026 Mar 30;19:34. doi: 10.1186/s13040-026-00550-4 (PMC13159293; doi:10.1186/s13040-026-00550-4)
Supplement: Supplementary file 1 — Supplementary Material 1 [file 13040_2026_550_MOESM1_ESM.pdf]

Additional file 1: Supplementary Information

For: A Biology-Based Quality-Diversity Algorithm for Drug Repurposing in Alzheimer's Disease Using Automated Machine Learning

Sisi Shao[1], Pedro Henrique Ribeiro[2], Alena Orlenko[2], Katie M. Cardone[3], Christina M. Ramirez[1], Li Shen[4,5], Marylyn D. Ritchie[3,4,5], Jason H. Moore[1,2*]

[1*]Department of Biostatistics, Fielding School of Public Health, University of California, Los Angeles, CA, USA.
[2]Department of Computational Biomedicine, Cedars-Sinai Medical Center, Los Angeles, CA, USA.
[3]Department of Genetics, University of Pennsylvania, Philadelphia, PA, USA.
[4]Institute for Biomedical Informatics, University of Pennsylvania, Philadelphia, PA, USA.
[5]Division of Informatics, Department of Biostatistics, Epidemiology and Informatics, University of Pennsylvania, Philadelphia, PA, USA.

*Corresponding author(s). E-mail(s): Jason.Moore@csmc.edu;

This supplementary document provides detailed mathematical formulations, architectural descriptions, and implementation settings for the proposed novelty-guided AutoML framework. It includes extended feature construction protocols, additional experimental results validating robustness across multiple embedding dimensions (including full 5-seed replications), and an extended mechanistic analysis of key candidates (Triclosan, Ketamine, and Quazepam) supported by recent 2024–2025 literature.

S1 Supplementary Methods

S1.1 GNN Architectures for Biologically-Informed Feature Representation

This section expands on the embedding generation process summarized in the main manuscript. The GNN architectures described here produce the embeddings visualized in Figures 1 and 2 of the main text.

S1.1.1 Foundations of Graph Neural Networks

Graph Neural Networks (GNNs) are a class of machine learning models specifically designed for graph-structured data [1]. Mathematically, a graph is defined as $G = (\mathcal{V}, \mathcal{E})$, where:

- $\mathcal{V}$ is the set of nodes (e.g., drugs, genes).
- $\mathcal{E}$ is the set of edges representing interactions.

Each node $i \in \mathcal{V}$ is associated with a feature vector $\mathbf{x}_i \in \mathbb{R}^F$. The graph structure is encoded in an adjacency matrix $\mathbf{A} \in \{0,1\}^{N \times N}$, where $N = |\mathcal{V}|$. The primary goal is to learn a low-dimensional embedding $\mathbf{z}_i \in \mathbb{R}^d$ for each node that captures both its intrinsic features and its local neighborhood context via message passing.

S1.1.2 GraphSAGE for Gene Embedding

For gene embeddings, we employed GraphSAGE [2]. GraphSAGE supports various aggregation strategies to combine information from neighbor nodes $\mathcal{N}(v)$. The general forms include:

General Aggregation Mechanisms

- **Mean Aggregator:**

$$h_v^{(l+1)} = \sigma\left(W^{(l)} \cdot \text{mean}\left(\{h_u^{(l)} : u \in \mathcal{N}(v)\}\right) + b^{(l)}\right)$$

- **LSTM Aggregator:**

$$h_v^{(l+1)} = \text{LSTM}\left(\{h_u^{(l)} : u \in \mathcal{N}(v)\}\right)$$

- **Pooling Aggregator:**

$$h_v^{(l+1)} = \text{pool}\left(\{h_u^{(l)} : u \in \mathcal{N}(v)\}\right)$$

Specific Implementation

In this study, we specifically employed the **Mean Aggregator** for its computational efficiency. The embedding for a node i at the k-th layer was computed as:

$$\mathbf{h}_i^{(k)} = \text{ReLU}\left(\mathbf{W}^{(k)} \cdot \text{MEAN}\left(\{\mathbf{h}_i^{(k-1)}\} \cup \{\mathbf{h}_j^{(k-1)} : j \in \mathcal{N}(i)\}\right)\right), \tag{S1}$$

where $\mathbf{W}^{(k)}$ is a learnable weight matrix. The final embedding is $\mathbf{z}_i = \mathbf{h}_i^{(K)}$ (with $K = 2$).

S1.1.3 Variational Graph Autoencoder (VGAE) for Drug Embedding

For drugs, we used a Variational Graph Autoencoder (VGAE) [3] to model the probabilistic nature of drug interactions.

Encoder

A two-layer GCN parameterizes the approximate posterior distribution $q(\mathbf{z}_i|\mathbf{X}, \mathbf{A})$:

$$\boldsymbol{\mu} = \text{GCN}_\mu(\mathbf{X}, \mathbf{A}), \quad \log \boldsymbol{\sigma} = \text{GCN}_\sigma(\mathbf{X}, \mathbf{A})$$

Latent Distribution and Sampling

The latent embedding $\mathbf{z}_i$ is sampled from a Gaussian distribution using the reparameterization trick to allow for backpropagation:

$$q(\mathbf{z}_i|\mathbf{X}, \mathbf{A}) \sim \mathcal{N}(\boldsymbol{\mu}_i, \text{diag}(\boldsymbol{\sigma}_i^2))$$

$$\mathbf{z}_i = \boldsymbol{\mu}_i + \boldsymbol{\sigma}_i \cdot \boldsymbol{\epsilon}, \quad \text{where } \boldsymbol{\epsilon} \sim \mathcal{N}(0, \mathbf{I})$$

Decoder

The decoder reconstructs the adjacency matrix edges via an inner product:

$$\hat{A}_{ij} = \sigma(\mathbf{z}_i^\top \mathbf{z}_j) \tag{S2}$$

S1.1.4 Training Objectives and Clustering Loss

A key innovation is the integration of a **clustering loss** to enforce biological coherence.

GraphSAGE Training Objective

The total loss function is composed of reconstruction, clustering, and regularization terms:

$$\mathcal{L}_{\text{SAGE}} = \mathcal{L}_{\text{recon}} + \lambda_{\text{cluster}}\mathcal{L}_{\text{cluster}} + \lambda_{\text{reg}}\mathcal{L}_{\text{reg}} \tag{S3}$$

where:

- **Reconstruction Loss (Binary Cross-Entropy):**

$$\mathcal{L}_{\text{recon}} = -\sum_{(i,j)\in\mathbf{E}} \left[A_{ij} \log \hat{A}_{ij} + (1 - A_{ij}) \log(1 - \hat{A}_{ij}) \right]$$

- **Clustering Loss:** Penalizes pairwise distances within the target set T of 101 known AD genes:

$$\mathcal{L}_{\text{cluster}} = \frac{1}{|T|} \sum_{i\in T} \max_{j\in T} \|\mathbf{z}_i - \mathbf{z}_j\|_2^2 \tag{S4}$$

- **Regularization Loss (L_2):**

$$\mathcal{L}_{\text{reg}} = \sum_{k=1}^{K} \|\mathbf{W}^{(k)}\|_2^2$$

VGAE Training Objective

The VGAE objective maximizes the evidence lower bound (ELBO), augmented with the clustering term:

$$\mathcal{L}_{\text{VGAE}} = \mathcal{L}_{\text{recon}} + \mathcal{L}_{\text{KL}} + \lambda_{\text{cluster}}\mathcal{L}_{\text{cluster}} \tag{S5}$$

where $\mathcal{L}_{\text{KL}}$ is the Kullback-Leibler divergence between the posterior and the prior $p(\mathbf{Z}) = \mathcal{N}(0, \mathbf{I})$:

$$\mathcal{L}_{\text{KL}} = \text{KL}\left(q(\mathbf{Z}|\mathbf{X}, \mathbf{A}) \| p(\mathbf{Z})\right) = \sum_{i=1}^{N} \text{KL}\left(q(\mathbf{z}_i|\mathbf{X}, \mathbf{A}) \| p(\mathbf{z}_i)\right)$$

Alternative Clustering Loss Formulation

While we utilized the max-distance formulation (Eq. S4), future refinements could utilize a ratio-based objective to minimize intra-cluster variance relative to global variance:

$$\mathcal{L}_{\text{cluster}}^{\text{alt}} = \frac{\frac{1}{|T|}\sum_{i\in T} \|\mathbf{z}_i - \bar{\mathbf{z}}_T\|_2^2}{\frac{1}{|V|}\sum_{j\in V} \|\mathbf{z}_j - \bar{\mathbf{z}}_V\|_2^2 + \epsilon}$$

S1.2 Feature Construction and Pipeline Evaluation

S1.2.1 Selection of Clustering Parameters (Sensitivity Analysis)

To determine the optimal number of clusters (k) for the drug feature construction, we employed the Elbow Method on the K-Means inertia. As shown in **Figure S1**, we iterated k from 2 to 100.

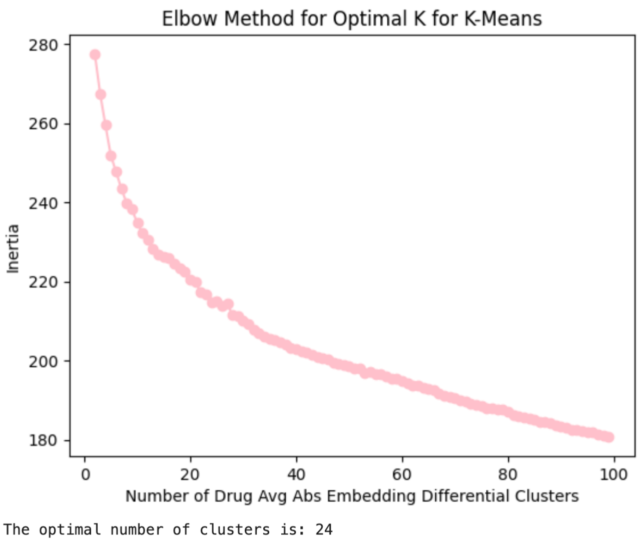

Fig. S1: **Sensitivity analysis for optimal k selection.** The plot shows the K-Means inertia (y-axis) decreasing as the number of clusters (x-axis) increases. The inflection point ("elbow") is observed at approximately $k = 24$, which was selected to balance cluster compactness with feature granularity.

Rationale for Outlier Filtering (The "20" Threshold).

We analyzed the degree distribution of drug-gene interactions in AlzKB (Median = 10; Mean = 36.33). We selected a threshold of **20 gene interactions** (twice the median), corresponding to the ∼**72nd percentile**. This rigorous filtering step isolates the top ∼28% of highly promiscuous super-connector drugs, preventing them from distorting the cluster centroids.

Justification for Clustering Loss Weight ($\lambda = 1$).

The weighting factor λ_{cluster} in Equations S3 and S5 controls the trade-off between preserving global graph topology ($\mathcal{L}_{\text{recon}}$) and enforcing local biological coherence ($\mathcal{L}_{\text{cluster}}$). We selected a fixed value of $\lambda = 1$ based on preliminary empirical observations indicating that both loss components operate within comparable numerical magnitudes (typically in the range of 10^{-1} to 10^{0}) during training. This unit weighting represents a deliberate inductive bias: we hypothesize that the biological prior (i.e., that known AD entities should cluster together) is equally as informative as the structural information derived from the knowledge graph. By setting $\lambda = 1$, we ensure that gradient updates are driven balancedly by both objectives, preventing the model from either ignoring biological constraints (if $\lambda \to 0$) or overfitting to clusters at the expense of feature richness (if $\lambda \gg 1$).

S1.2.2 Biological Validation of GNN Embeddings

To ensure that the learned GNN embeddings capture meaningful pharmacological relationships rather than random noise, we performed an external validation using the Anatomical Therapeutic Chemical (ATC) Classification System. We extracted ATC level-4 codes for all drugs in AlzKB and categorized pairwise relationships into two groups: "Same Class" (pairs sharing the same code) and "Different Class" (disjoint pairs).

We formulated the following statistical hypotheses:

- **Null Hypothesis (H_0):** There is no difference in the distribution of Euclidean embedding distances between drug pairs in the same ATC class versus those in different classes ($\mu_{\text{same}} = \mu_{\text{diff}}$).
- **Alternative Hypothesis (H_1):** Drug pairs within the same ATC class have smaller Euclidean distances than those in different classes ($\mu_{\text{same}} < \mu_{\text{diff}}$), indicating biological clustering.

We conducted a one-sided **Mann-Whitney U test** to compare the distributions. As shown in **Figure S2**, the mean Euclidean distance for same-class pairs was **0.867**, compared to **0.924** for different-class pairs. The test yielded a statistically significant difference with a p-value of 8.90×10^{-61}, leading to the rejection of H_0. This confirms that our GNN architecture, supervised by the clustering loss, successfully encoded functional biological similarities into the latent space.

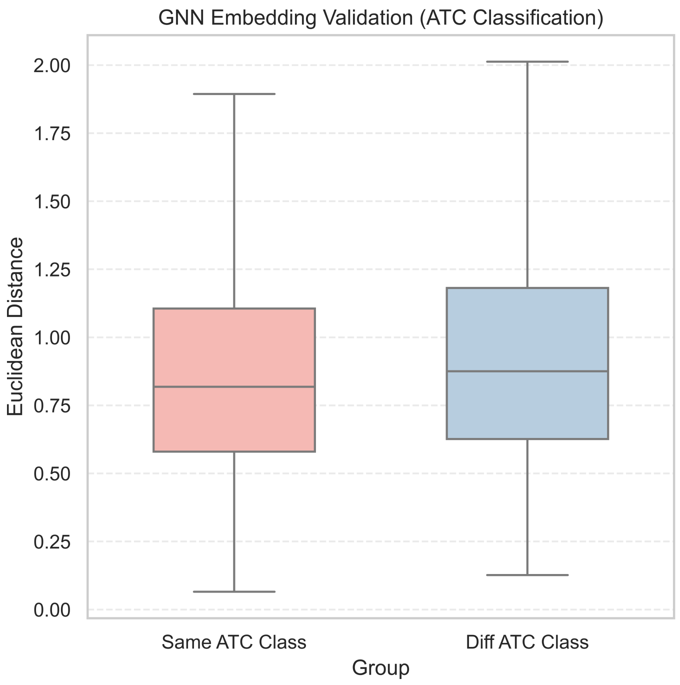

Fig. S2: **Validation of GNN embeddings against ATC classification.** Pairwise Euclidean distances between drugs sharing the same ATC code (green) are consistently lower than those between disjoint pairs (orange). A Mann-Whitney U test confirms a statistically significant separation between the distributions ($p < 0.001$), supporting the biological relevance of the learned embedding space.

S1.2.3 Drug Similarity Metrics

We computed three distances between GNN drug embeddings ($\mathbf{z} \in \mathbb{R}^d$) to construct the MAP-Elites novelty dimensions:

Euclidean Distance

A conventional measure of geometric distance in the embedding space.

$$D_{ij}^{\text{Euclidean}} = \|\mathbf{z}_i - \mathbf{z}_j\|_2 = \sqrt{\sum_{k=1}^{d} (\mathbf{z}_{ik} - \mathbf{z}_{jk})^2} \tag{S6}$$

Canberra Distance

A weighted metric that emphasizes relative differences between vectors, making it sensitive to small changes.

$$D_{ij}^{\text{Canberra}} = \sum_{k=1}^{d} \frac{|\mathbf{z}_{ik} - \mathbf{z}_{jk}|}{|\mathbf{z}_{ik}| + |\mathbf{z}_{jk}|} \tag{S7}$$

Cosine Distance

A metric that captures the orientation of embedding vectors rather than their magnitude.

$$D_{ij}^{\text{Cosine}} = 1 - \frac{\mathbf{z}_i \cdot \mathbf{z}_j}{\|\mathbf{z}_i\|_2 \|\mathbf{z}_j\|_2} \tag{S8}$$

S1.2.4 Gene Score Calculation

For each individual in the ADSP cohort, a score for each gene was computed as:

$$\text{Gene Score} = \frac{\sum(\beta \times \text{Genotype})}{n_{\text{variants}}} \tag{S9}$$

where β is the GWAS log odds ratio, *Genotype* is the allele dosage (0, 1, or 2), and n_{variants} is the variant count.

S1.3 Supplementary Analysis of Embedding Topology

S1.3.1 Biological Interpretation of Embedding Arms (Figure 1a)

To validate that the topological structure of the learned gene embeddings (Main Manuscript, Figure 1a) reflects meaningful biological heterogeneity rather than arbitrary artifacts, we performed a post-hoc enrichment analysis on the genes located at the extremes of the UMAP embedding space. Our analysis reveals that the embedding space disentangles three distinct pathophysiological axes of Alzheimer's Disease:

- **Left Arm (Amyloid Processing & Neuroprotection):** The genes with the lowest dimension-1 coordinates include key drivers of AD pathology, such as the amyloid precursor protein (*APP*) and alpha-secretase (*ADAM10*), alongside neuroprotective and vascular markers like *VEGFA* and *NOS3*. This localizes the core molecular pathology within this region.
- **Right Arm (Ion Homeostasis & Transport):** The genes with the highest dimension-1 coordinates are heavily enriched for ion channels (e.g., *CACNA1H*, *KCNMB1*, *HCN3*, *SCN4A*) and solute carriers. This indicates a module representing membrane excitability and physiological transport, distinct from the core amyloidogenic processes.
- **Top Arm (Neuroinflammation & TNF Signaling):** Examining the genes with the highest dimension-2 coordinates reveals a distinct cluster enriched for immune regulators, particularly members of the TNF superfamily (e.g., *LTA*, *LTB*,

TNFSF14, *LTBR*) and complement factors (*CFD*). This suggests the model successfully disentangled the neuroinflammatory axis of AD from the amyloid and synaptic axes.

S1.3.2 Full List of Genes in Topological Arms

The top 20 genes identified in each topological arm (based on extreme UMAP coordinates) are listed below. Bolded genes highlight key functional markers discussed above.

Left Arm Genes (Core Pathology): FGFR3, TPI1, ITGB1, TF, ***VEGFA****, HMOX1, PGRMC1, ARRB1,* ***NOS3****, ESR1, NCF4, DPYSL2,* ***APP****, CRABP2,* ***ADAM10****, ZAP70, PTPN6, PTPN11, KDM4C, CD79B.*
Right Arm Genes (Ion/Transport): GPNMB, GSTA1, ATP6V1B2, GDF2, ***SLC22A2****, PTH, PIP, KDM6A, CD244, FBP2, NEU3,* ***SCN4A****, EMP1,* ***CACNA1H****,* ***KCNMB1****,* ***KCNJ13****,* ***SLC5A7****, KIR2DS1,* ***HCN3****, CELA1.*
Top Arm Genes (Neuroinflammation): ***LTA****, PAPOLA,* ***LTB****, CLK3, TSSK1B,* ***LTBR****,* ***CFD****, SRMS, NPY5R, CDK17, SCAP, SLC22A5,* ***AXL****,* ***TNFSF14****,* ***CISH****.*

S1.3.3 Ablation Study: Impact of Clustering Loss

To demonstrate the necessity of our proposed domain-specific clustering loss ($\mathcal{L}_{cluster}$), we conducted an ablation study where the models were trained using only the reconstruction link prediction loss, omitting the clustering term.

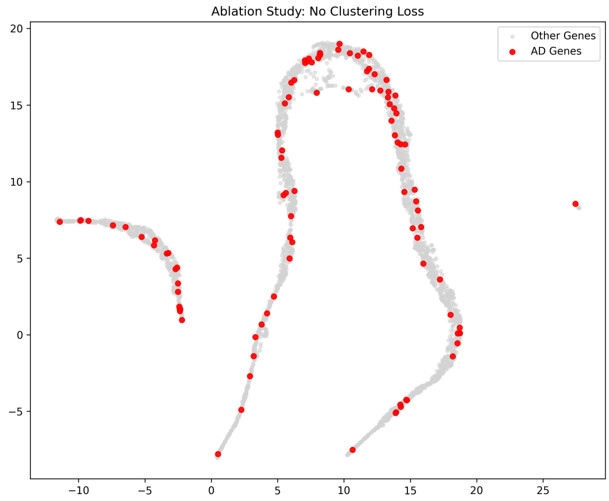

Fig. S3: **Ablation Study: Gene Embeddings (Without Clustering Loss).** UMAP visualization of gene embeddings trained without the biological clustering loss. Unlike the main model (Figure 1), known AD genes (red points) are scattered throughout the embedding space rather than forming a cohesive cluster. This demonstrates that the cohesive AD cluster observed in the main manuscript is explicitly driven by our loss function design.

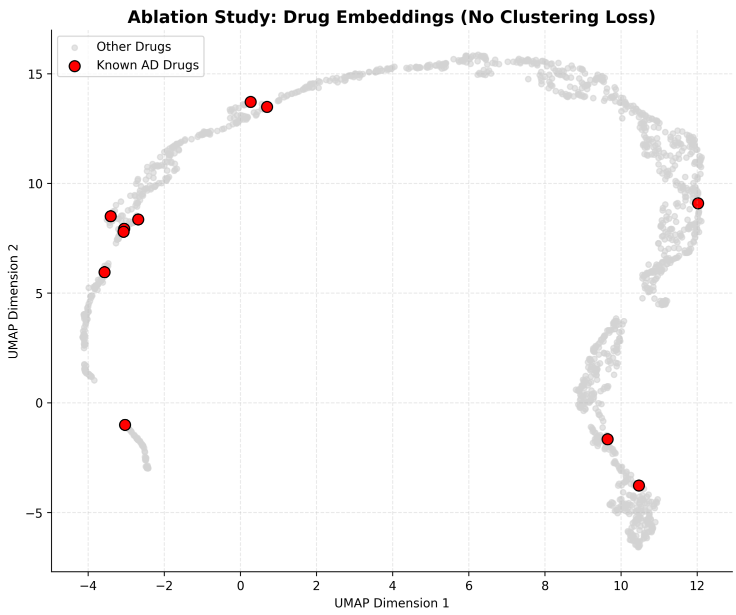

Fig. S4: **Ablation Study: Drug Embeddings (Without Clustering Loss).** UMAP visualization of drug embeddings trained without the biological clustering loss. Note that the UMAP projection scale is independent from the gene embeddings. Similar to the gene results, known FDA-approved AD drugs (red points) are widely scattered across the latent space. This explicitly demonstrates that the cohesive AD drug neighborhoods observed in our main analyses are driven by the custom clustering loss design.

S2 Supplementary Experimental Results

S2.1 Robustness Across Independent Random Seeds

To rigorously validate reproducibility, we visualize the MAP-Elites archival maps from all 5 independent experimental runs.

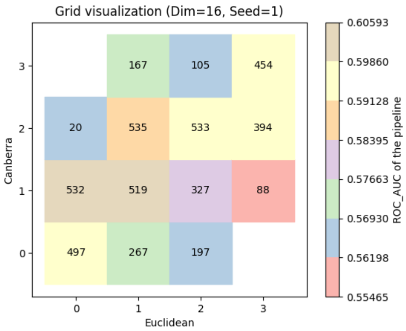

(a) Seed 1 (Triclosan found)

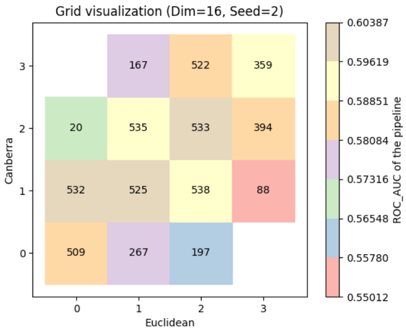

(b) Seed 2

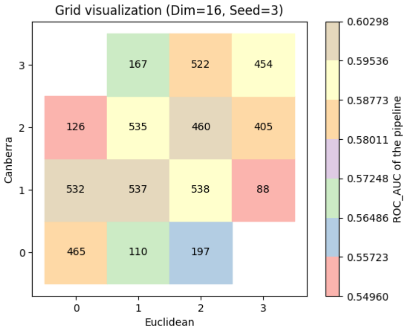

(c) Seed 3 (Triclosan found)

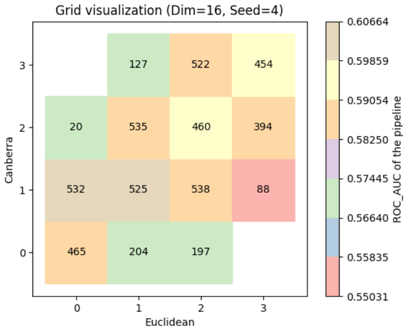

(d) Seed 4 (Triclosan found)

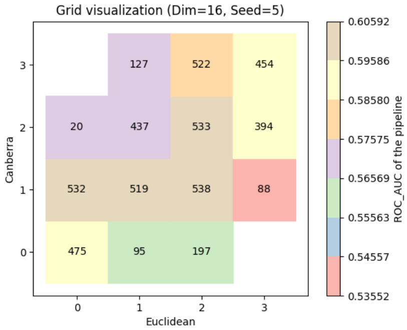

(e) Seed 5 (Triclosan found)

Fig. S5: **Robustness of Grid Exploration (Dim=16).** The search consistently populates high-novelty regions (e.g., cell 3,3), confirming that the identification of **Triclosan** is robust.

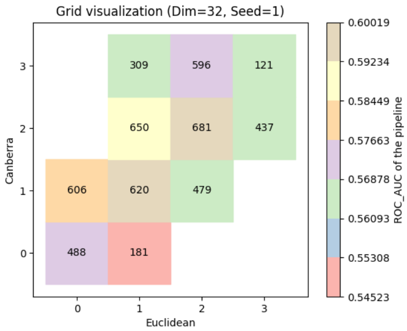

(a) Seed 1

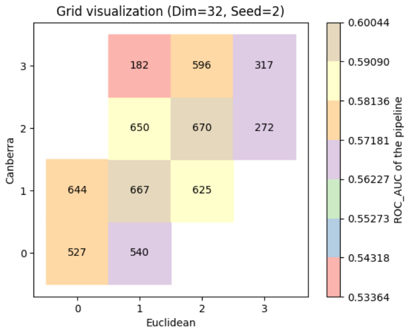

(b) Seed 2

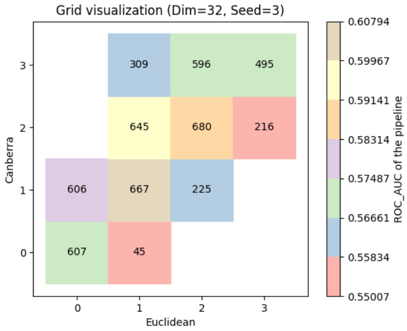

(c) Seed 3

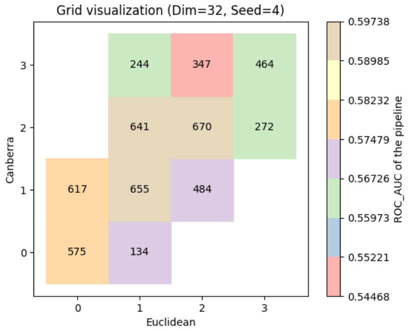

(d) Seed 4 (Quazepam + Ketamine found)

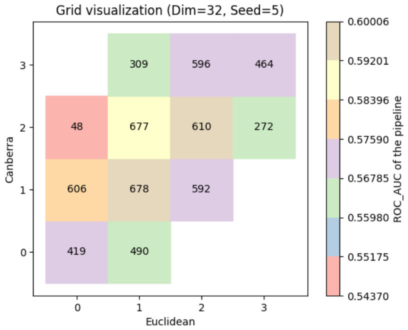

(e) Seed 5 (Quazepam + Ketamine found)

Fig. S6: **Robustness of Grid Exploration (Dim=32).** Notable recovery of the **Ketamine** and **Quazepam** feature set in Seeds 4 and 5.

S2.2 Gene Ontology (GO) Network Analysis

We visualize GO networks for the novel candidates identified in our exploratory analysis to confirm that their extracted feature sets form coherent biological modules. The GO enrichment network analysis was conducted using the Harmonizome tool [4]. In these visualizations, nodes represent individual genes from the feature set, and edges denote shared Gene Ontology biological processes. Network modules (distinct functional clusters) were identified using Harmonizome's default force-directed layout and community detection algorithms.

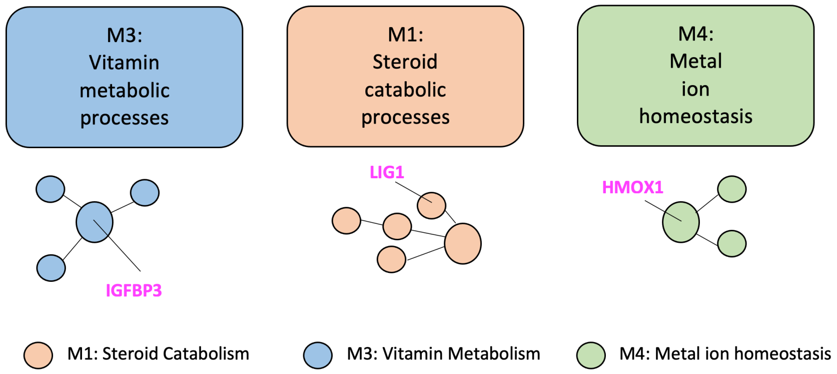

Fig. S7: **GO-based network clustering (Dim=16, Exemestane Run).** Functional modules enriched for steroid catabolism (M1) and vitamin metabolism (M3), corresponding to the mechanism of **Exemestane**. The network partitioned into 3 major functional modules, cleanly separating distinct metabolic pathways.

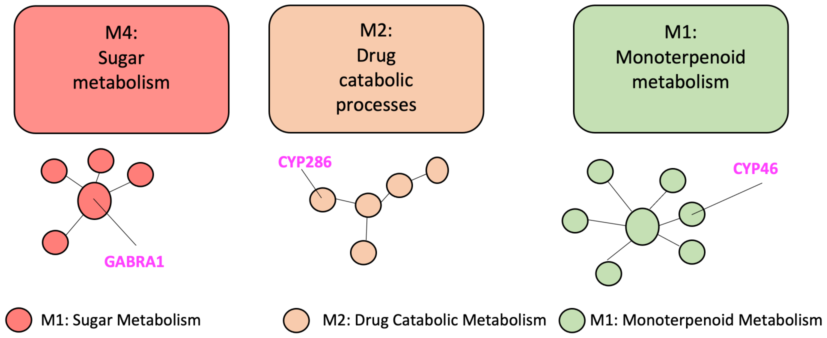

Fig. S8: **GO-based network clustering (Dim=32, Felodipine Run).** Clustering of ion channel regulation genes (e.g., GABRA1) corresponding to **Felodipine**. The network visualization highlights 4 distinct functional modules, prominently featuring a dense module dedicated to neurotransmitter receptor activity.

S2.3 Analysis of Additional MAP-Elites Cells

We distinguish between candidates found during the initial exploratory phase and those robustly validated in specific seeds (Table S1).

S2.4 Extended Mechanistic Analysis of Candidates

We expand on the mechanistic implications of three key candidates: Triclosan, Ketamine, and Quazepam, incorporating recent evidence from 2024–2025.

S2.4.1 Triclosan: Environmental Risk Factor

Triclosan, a widely used antimicrobial, has recently been identified as a potential environmental risk factor for AD. Cheng et al. (2025) integrated machine learning with Mendelian randomization to reveal that Triclosan exposure disrupts critical AD-associated genes (e.g., *APP*, *DRD2*) [5]. Furthermore, exposome-wide studies in US older adults have linked serum Triclosan levels to cognitive decline [6]. Mechanistically, Triclosan induces neuroinflammation and necroptosis by upregulating TNF-α and activating the TNFR1 signaling axis, leading to RIPK1/RIPK3/MLKL-mediated cell death [7]. These findings align with our model's identification of Triclosan-associated gene sets enriched in inflammatory pathways.

S2.4.2 Ketamine: NMDA Antagonism and Synaptic Plasticity

Ketamine, a non-competitive NMDA receptor antagonist, shows promise for treating AD-associated depression and potentially improving cognition. A 2025 systematic review highlight its efficacy in overcoming treatment-resistant depression in AD patients, possibly via synaptic plasticity enhancement [8]. Recent case reports document rapid improvements in cognition and daily functioning following ketamine therapy [9]. Moreover, its metabolite (2R,6R)-hydroxynorketamine (HNK) has been shown to rescue hippocampal protein synthesis and memory in AD mouse models [10]. However, some studies in 5XFAD mice report no reduction in amyloid plaque load [11], suggesting its benefits may be symptom-specific or stage-dependent.

S2.4.3 Quazepam: GABAergic Modulation and E/I Balance

The robust co-retrieval of **Quazepam** (a long-acting benzodiazepine) with Ketamine in our high-dimensional search (Dim=32, Seeds 4/5) suggests a model preference for restoring the **Excitation/Inhibition (E/I) balance**.

GABAergic Mechanisms

By acting as a $GABA_A$ agonist, Quazepam may counteract the neuronal hyperexcitability characteristic of early AD. Preclinical studies support this "neuroprotective" hypothesis in specific contexts: low-dose diazepam improved cognition and reduced amyloid load in APP/PS1 mice by modulating AMPA receptors [12], and midazolam inhibited amyloid fibril formation in vitro [13].

Clinical Complexity and Risks

However, the clinical translation of benzodiazepines is complex. Epidemiological evidence is mixed: some studies link long-term use to increased dementia risk [14], while others find no causal association after adjusting for protopathic bias [15, 16]. In established AD, benzodiazepines are generally avoided due to risks of delirium and falls [17]. Furthermore, chronic use may mechanistically exacerbate pathology by upregulating amyloid-β via TSPO suppression [18] or promoting tau phosphorylation [19].

Conclusion

Our findings do not recommend Quazepam monotherapy but rather validate the algorithm's ability to detect potent modulators of the AD-associated GABAergic network. The simultaneous selection of an NMDA antagonist (Ketamine) and a GABA agonist (Quazepam) highlights a "network-balancing" therapeutic hypothesis.

S2.5 Lack of Reproducibility in Standard Evolution (NSGA-II)

To visually demonstrate why standard multi-objective evolution is insufficient for consistent candidate retrieval, we plot the archival maps of NSGA-II (Dim 16) across 5 independent random seeds. As shown in **Figure S9**, NSGA-II identified Triclosan in only 1 out of 5 seeds (Seed 3). This highlights the severe stochasticity of the baseline method compared to the highly reproducible discovery enabled by MAP-Elites (Figure S5).

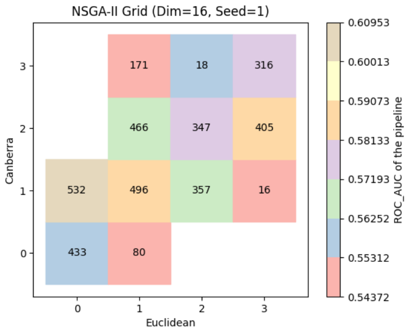

(a) Seed 1 (Missed)

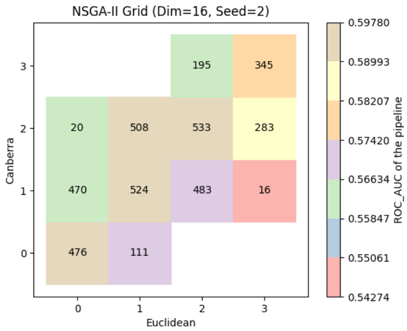

(b) Seed 2 (Missed)

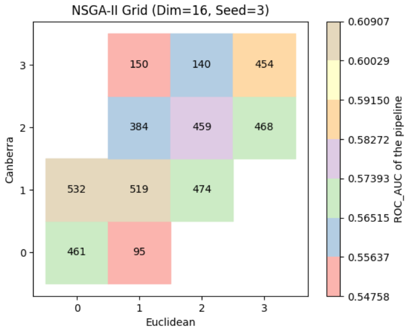

(c) Seed 3 (Triclosan found)

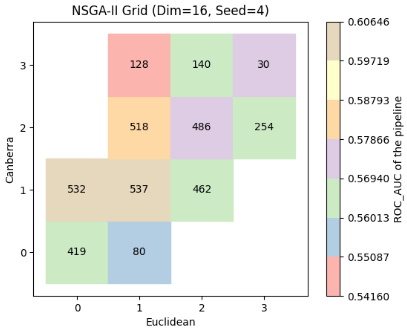

(d) Seed 4 (Missed)

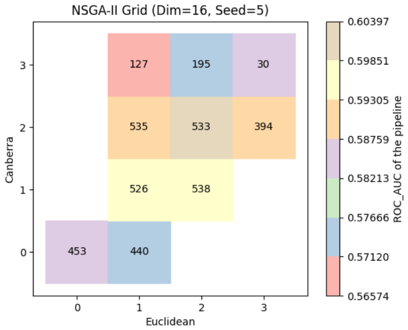

(e) Seed 5 (Missed)

Fig. S9: **Archival maps for NSGA-II in Dim 16 across 5 independent seeds.** Without explicit novelty preservation, NSGA-II is highly stochastic and only identified Triclosan in 1 out of 5 runs (Seed 1).

S2.6 Complete Loss of Niche Candidates in High Dimensions (NSGA-II)

The algorithmic stochasticity of standard multi-objective evolution is further exacerbated in high-dimensional search spaces. To demonstrate this, we plot the archival maps of NSGA-II (Dim 32) across the 5 independent random seeds. As shown in **Figure S10**, NSGA-II completely failed to identify the highly novel Ketamine/Quazepam pair in all 5 runs (0/5). Without the explicit Quality-Diversity preservation mechanism employed by MAP-Elites, the unguided Pareto front in NSGA-II suffers from severe directional loss, failing to protect mechanically unique but globally suboptimal solutions in complex environments.

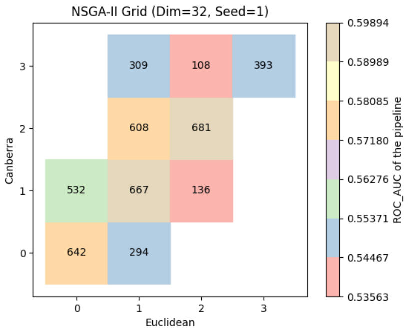

(a) Seed 1 (Missed)

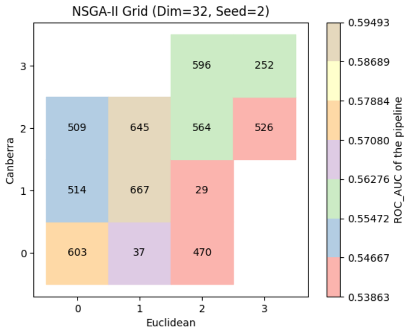

(b) Seed 2 (Missed)

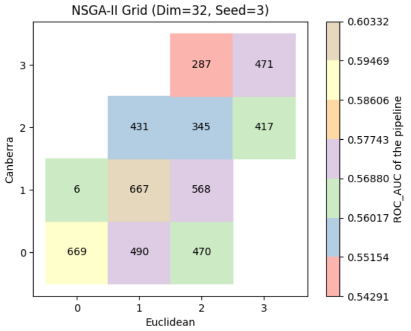

(c) Seed 3 (Missed)

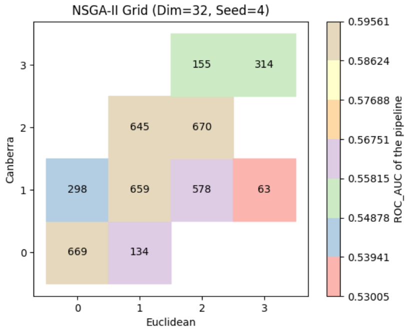

(d) Seed 4 (Missed)

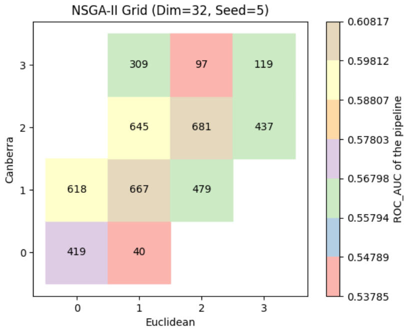

(e) Seed 5 (Missed)

Fig. S10: **Archival maps for NSGA-II in Dim 32 across 5 independent seeds.** NSGA-II completely failed to identify the Quazepam + Ketamine feature set across all independent runs, demonstrating severe capability loss in high-dimensional feature spaces.

S3 Computational Complexity and Runtime Analysis

To rigorously assess scalability, we tracked the wall-clock time for 20 independent jobs (5 seeds per method per dimension) executed on the high-performance computing cluster.

Hardware Specification

We utilized a GPU-accelerated node equipped with **Intel Xeon Gold 6254 CPUs (18 cores, 36 threads)**, **768 GB of RAM**, and **NVIDIA Tesla V100-SXM2 (32GB)** GPUs.

Table S2 details the runtime comparison.

1. **Scalability:** While NSGA-II slowed down by ∼33% in high dimensions (Dim 32), MAP-Elites maintained consistent performance.
2. **Efficiency:** In Dim 32, MAP-Elites was **∼48% faster** than NSGA-II.

S4 Extended Clinical Background and Rationale

S4.1 The Current Landscape of AD Therapeutics

Alzheimer's disease (AD) is a progressive neurodegenerative disorder affecting millions, with high clinical and economic burdens. Despite decades of intensive research, developing broadly effective disease-modifying treatments has proven exceptionally challenging [20]. While recent anti-amyloid therapies like Lecanemab and Donanemab mark significant progress, they offer modest clinical benefits and are associated with notable safety concerns, highlighting the need for further innovation [21, 22]. The established therapeutic landscape includes drugs such as donepezil and memantine, which provide only symptomatic relief, and controversial treatments like aducanumab with limited efficacy [23]. Given the high failure rate of novel AD therapeutics and the lengthy, costly development process, alternative therapeutic strategies are urgently needed [24].

S4.2 The Strategic Value of Drug Repurposing

Drug repurposing—finding new therapeutic uses for existing drugs—offers a faster and more cost-effective approach to AD treatment. Leveraging established safety profiles reduces the risk of unexpected adverse events, while preclinical data can accelerate clinical translation and streamline regulatory approval [25, 26].

The landscape of AD therapeutics increasingly recognizes the potential of drug repurposing. As of early 2024, repurposed agents constitute a significant portion of the AD drug development pipeline, with 39 such agents under investigation across various clinical trial phases [27]. These repurposed candidates are diverse, targeting different mechanisms; for example, they make up 40% of disease-modifying small molecules and 40% of cognitive-enhancing agents in the current pipeline [27].

Advanced computational strategies and the analysis of multi-omics data are pivotal in identifying such candidates. For instance, bumetanide has been nominated based

on transcriptomic studies, while analysis of electronic health records has pointed to drugs like sildenafil and telmisartan as having repurposing potential for AD [28]. Other computational approaches, including clinical trial emulation, have identified agents such as pantoprazole and fluticasone as promising avenues for further investigation [28]. These examples highlight the active and varied efforts to leverage existing drugs to address the multifaceted nature of AD. Notably, resources like the Alzheimer's Knowledge Base (AlzKB) integrate heterogeneous biomedical data to enable graph-based inference of novel therapeutic hypotheses [20].

References

[1] Wu Z, Pan S, Chen F, Long G, Zhang C, Philip SY. A comprehensive survey on graph neural networks. IEEE transactions on neural networks and learning systems. 2020;32(1):4–24.

[2] Hamilton W, Ying Z, Leskovec J. Inductive representation learning on large graphs. Advances in neural information processing systems. 2017;30.

[3] Kipf TN, Welling M. Variational graph auto-encoders. arXiv preprint arXiv:161107308. 2016;.

[4] Rouillard AD, Gundersen GW, Fernandez NF, Wang Z, Monteiro CD, McDermott MG, et al. The harmonizome: a collection of processed datasets gathered to serve and mine knowledge about genes and proteins. Database. 2016;2016:baw100.

[5] Cheng F, Gao H, Yan B, Chen F, Lei P. From disinfectant to neurodegeneration: Integrating machine learning and mendelian randomization reveals triclosan as a novel environmental risk factor for Alzheimer's disease. Environmental Pollution. 2025;385:127068.

[6] Jang H, Lee J, Nguyen VK, Shin HM. Exposome-wide association study of cognitive function in US older adults using the NHANES data. Exposome. 2025;5(1):osaf001.

[7] Asimakidou E, Reynolds R, Barron AM, Lo CH. Autolysosomal acidification impairment as a mediator for TNFR1 induced neuronal necroptosis in Alzheimer's disease. Neural Regeneration Research. 2024;19(9):1869–1870. https://doi.org/10.4103/1673-5374.390979.

[8] Altamura M, Leccisotti I, Moretti MC, Bellomo A, Panza F, Cassano T, et al. Can ketamine therapy overcome treatment-resistant depression in Alzheimer's disease and older adults? Preclinical and clinical evidence. Biomedicine & Pharmacotherapy. 2025 Jul;188:118199. https://doi.org/10.1016/j.biopha.2025.118199.

[9] Tadros M, Rente Lavastida D, Hanna A. Therapeutic Potential of Intravenous Ketamine in Early-Onset Dementia: A Case Report. Cureus. 2024

Jul;16(7):e65261. https://doi.org/10.7759/cureus.65261.

[10] Ribeiro FC, et al. The ketamine metabolite (2R,6R)-hydroxynorketamine rescues hippocampal mRNA translation, synaptic plasticity and memory in mouse models of Alzheimer's disease. Alzheimer's & Dementia. 2024;20(8):5398–5410.

[11] Wright AL, Weible AP, Estes OB, Wehr M. Ketamine does not rescue plaque load or gap detection in the 5XFAD mouse model of Alzheimer's disease. Frontiers in Aging Neuroscience. 2025 Feb;17:1505908. https://doi.org/10.3389/fnagi.2025.1505908.

[12] Chen J, Zhang M, Shen Z, Tang M, Zeng Y, Bai D, et al. Low-dose diazepam improves cognitive function in APP/PS1 mouse models: Involvement of AMPA receptors. Brain Research. 2024;1845:149207. https://doi.org/10.1016/j.brainres.2024.149207.

[13] Yamamoto N, Arima H, Sugiura T, Ikeda K. Midazolam inhibits the formation of amyloid fibrils and GM1 ganglioside-rich microdomains in presynaptic membranes through the $GABA_A$ receptor. Biochemical and Biophysical Research Communications. 2015;457(4):547–553. https://doi.org/10.1016/j.bbrc.2015.01.022.

[14] Billioti de Gage S, Moride Y, Ducruet T, Kurth T, Verdoux H, Tournier M, et al. Benzodiazepine use and risk of Alzheimer's disease: case-control study. BMJ. 2014;349:g5205. https://doi.org/10.1136/bmj.g5205.

[15] Tysinger B, Zissimopoulos J, Joyce, Geoffrey, et al. Benzodiazepine use and the risk of dementia: evidence from Medicare claims and case-control analyses. Alzheimer's & Dementia: Translational Research & Clinical Interventions. 2022;8(1):e12242. https://doi.org/10.1002/trc2.12242.

[16] Defrancesco M, Marksteiner J, Fleischhacker WW, Blasko I. Use of benzodiazepines in Alzheimer's disease: a systematic review of literature. Int J Neuropsychopharmacol. 2015;18(10):pyv055. https://doi.org/10.1093/ijnp/pyv055.

[17] Dyer AH, Murphy C, Lawlor B, Kennelly SP, Group NS. Cognitive outcomes of long-term benzodiazepine and related drug (BDZR) use in people living with mild to moderate Alzheimer's disease: results from NILVAD. Journal of the American Medical Directors Association. 2020;21(2):194–200. https://doi.org/10.1016/j.jamda.2019.08.006.

[18] Jung ME, Metzger DB, Hall J. Long-term, but not short-term, use of benzodiazepine impairs motor function and upregulates amyloid-β in part through the suppression of translocator protein. Pharmacology Biochemistry and Behavior. 2020;191:172873. https://doi.org/10.1016/j.pbb.2020.172873.

[19] Cheheltanan M, Petry F, Poitras I, Morin F, Planel E, Whittington RA. Administration of the benzodiazepine midazolam increases tau phosphorylation in the mouse brain. Neurobiology of Aging. 2019;75:11–24. https://doi.org/10.1016/j.neurobiolaging.2018.10.027.

[20] Romano JD, Truong V, Kumar R, Venkatesan M, Graham BE, Hao Y, et al. The Alzheimer's Knowledge Base: A Knowledge Graph for Alzheimer Disease Research. Journal of Medical Internet Research. 2024;26:e46777.

[21] Van Dyck CH, Swanson CJ, Aisen P, Bateman RJ, Chen C, Gee M, et al. Lecanemab in early Alzheimer's disease. New England Journal of Medicine. 2023;388(1):9–21.

[22] Sims JR, Zimmer JA, Evans CD, Lu M, Ardayfio P, Sparks J, et al. Donanemab in early symptomatic Alzheimer disease: the TRAILBLAZER-ALZ 2 randomized clinical trial. Jama. 2023;330(6):512–527.

[23] Passeri E, Elkhoury K, Morsink M, Broersen K, Linder M, Tamayol A, et al. Alzheimer's disease: treatment strategies and their limitations. International journal of molecular sciences. 2022;23(22):13954.

[24] Cummings JL, Morstorf T, Zhong K. Alzheimer's disease drug-development pipeline: few candidates, frequent failures. Alzheimer's research & therapy. 2014;6:1–7.

[25] Zhan P, Yu B, Ouyang L. Drug repurposing: An effective strategy to accelerate contemporary drug discovery. Drug discovery today. 2022;27(7):1785.

[26] Pushpakom S, Iorio F, Eyers PA, Escott KJ, Hopper S, Wells A, et al. Drug repurposing: progress, challenges and recommendations. Nature reviews Drug discovery. 2019;18(1):41–58.

[27] Cummings J, Zhou Y, Lee G, Zhong K, Fonseca J, Cheng F. Alzheimer's disease drug development pipeline: 2024. Alzheimer's & Dementia: Translational Research & Clinical Interventions. 2024;10(2):e12465.

[28] Cummings JL, Zhou Y, Van Stone A, Cammann D, Tonegawa-Kuji R, Fonseca J, et al. Drug repurposing for Alzheimer's disease and other neurodegenerative disorders. Nature Communications. 2025;16(1):1755.

Table S1: Supplementary MAP-Elites cells and associated genes.

Dim	Cell	Drug(s)	Genes
		**Phase 1: Initial Exploratory Analysis (Seeds Unspecified)**	
16	(2,3)	Digitoxin	CYP11A1, SLCO1A2, CYP3A4, ABCB1, ATP1A1, ALB, SLCO4C1, BRD4, ATF3, SRSF10, SETD2, PHKG2, CDC42, MDM2, CDK5R1, JUN, FOSL1, PPM1D, NR4A1, IGF2BP2, PMAIP1, NFKBIB, NFE2L2, FOS, LGALS8, DUSP6, FOXO3, CDK6, RIT1, CBLB, CDK7, CDC25A, CDK17, RGS2, OGA, TEX10, ICAM1, COL11A1, SMAD3, PSMD9, DYRK3, PKN2, CRKL, NCOA3, HIPK3, NFKB2, PTPN12, NFKBIA, PER1, CDKN1A, ATP1B1, DYRK1A, SIK3, PRKAA1, GABPA, BIRC2, NR3C1, CXCL2, NRAS, CLK4, CHD1, GADD45A, PTGS2, EGR1, DDIT3, EGFR, JUNB, NPC1, ADO, RPA1, PCNA, DPYSL2, CBR3, PGM1, LIPA, ATIC, ERBB2, KEAP1, HSPA8, IDH1, TUBB6, PSMD4, UMPS, CSK, EIF4EBP1, SPR, PSMB8, ALDH3A1, PPA2, PIK3C2B, IRAK1, CRYZ, CBR1, MPI, APEX1, SUV39H1, ICAM3, AKT1, CHEK2, TYMS, G6PD, TP53, RRP1B, PARP1, AKR7A2, HSPA1A, EPRS1, PLK1, PYGL, SACM1L, CCNA2, AKR1B1, RNASEH2A, SIRT5, CDC25B, CTSL, XBP1, BRCA1, TDP1, HSPB1
16	(3,2)	Medroxyprogesterone acetate + Sevoflurane	SHBG, AKR1C3, PGR, GPER1, NFKBIA, HSD3B2, PCNA, CYP3A4, CYP2C9, CDK4, CYP2C8, RGS2, ESR1, MYC, FOXO3, NR3C1, AR, ATP2C1, CYP2A6, GABRG1, GABRB1, GRIA1, GABRA6, CYP2E1, GABRQ, GABRB3, KCNK2, KCNK3, ALB, CYP2B6, ATP5F1D, GABRP, GABRA2, KCNA1, GABRA5, GLRA1, GABRD, GABRG3, GABRB2, GABRE, GABRA3, KCNK18, GABRA1, GABRG2, GABRA4, KCNK9, KCNK10
32	(2,3)	Budesonide	CYP3A4, NR3C1, TGFBR2, COL11A1, PAK1, FKBP5, CDKN1A, RGS2, PRKAG2, PPARG, MUC1, NFKBIA, PRKCD, SRPRB, PGM1, CASP7, FOXO3, CTSD, SERPINE1, TSC22D3, HSPB1, SIK1, DUSP1, PER1, MYC, PROS1, SMAD3, SQSTM1, AURKB, VIM, IL1B, TYMS, FHL2, FOSL1, HSPA1A, CCL2, GDF15, CD83, CAST, IGFBP3, DNMT1, LIF, PIN1
32	(3,2)	Rimonabant + Metixene	TYMS, CYP2C9, HP, CYP3A4, CYP2D6, CNR1, CNR2, MMP1, CYP1A2, HTR2C, CDK6, CHRM3, CHRM4, CHRM1, CHRM2, PSMB8, CHRM5
		**Phase 2: Robustness Validation (Independent Seeds 4 & 5)**	
**32**	**Seeds 4/5**	**Quazepam + Ketamine**	**Significant co-retrieval:** This pair suggests a mechanistic convergence on modulating the Excitation/Inhibition (E/I) balance. Ketamine targets NMDA receptors (glutamatergic), while Quazepam targets $GABA_A$ receptors (GABAergic).

Table S2: **Runtime comparison stratified by embedding dimension.** Mean ± SD wall-clock time (hours) across 5 independent seeds.

Method	Dimension	Generations	Time (h)	Efficiency Note
**MAP-Elites (Ours)**	16	500	**3.49 ± 0.44**	Consistent performance.
**MAP-Elites (Ours)**	32	500	**3.20 ± 0.48**	**Highly efficient in high-dim.**
NSGA-II (Baseline)	16	500	4.66 ± 1.03	Slower baseline.
NSGA-II (Baseline)	32	500	6.20 ± 0.45	Significant slowdown observed.
